# Correlative geochemical imaging of *Desmophyllum dianthus* reveals biomineralisation strategy as a key coral vital effect

**DOI:** 10.1038/s41598-024-61772-2

**Published:** 2024-05-15

**Authors:** Christopher D. Standish, Jacob Trend, Jacob Kleboe, Thomas B. Chalk, Sumeet Mahajan, J. Andy Milton, Tessa M. Page, Laura F. Robinson, Joseph A. Stewart, Gavin L. Foster

**Affiliations:** 1 School of Ocean and Earth Sciences, National Oceanography Centre, University of Southampton, European Way, Southampton, SO14 3ZH UK; 2 Department of Chemistry and Institute for Life Sciences, University of Southampton, Highfield Campus, University Road, Southampton, SO17 1BJ UK; 3 Aix Marseille Université, CNRS, IRD, INRAE, Coll France, CEREGE, Aix-en-Provence, France; 4 School of Earth Sciences, University of Bristol, Queens Road, Bristol, BS8 1RJ UK

**Keywords:** Biogeochemistry, Ocean sciences

## Abstract

The chemical and isotopic composition of stony coral skeletons form an important archive of past climate. However, these reconstructions are largely based on empirical relationships often complicated by “vital effects” arising from uncertain physiological processes of the coral holobiont. The skeletons of deep-sea corals, such as *Desmophyllum dianthus*, are characterised by micron-scale or larger geochemical heterogeneity associated with: (1) centres of calcification (COCs) where nucleation of new skeleton begins, and (2) fibres that thicken the skeleton. These features are difficult to sample cleanly using traditional techniques, resulting in uncertainty surrounding both the causes of geochemical differences and their influence on environmental signals. Here we combine optical, and in-situ chemical and isotopic, imaging tools across a range of spatial resolutions (~ 100 nm to 10 s of μm) in a correlative multimodal imaging (CMI) approach to isolate the microstructural geochemistry of each component. This reveals COCs are characterised by higher organic content, Mg, Li and Sr and lower U, B and δ^11^B compared to fibres, reflecting the contrasting biomineralisation mechanisms employed to construct each feature. CMI is rarely applied in Environmental/Earth Sciences, but here we illustrate the power of this approach to unpick the “vital effects” in *D. dianthus*, and by extension, other scleractinian corals.

## Introduction

Tropical coral reefs are the oceans’ most diverse ecosystems, they are home to millions of species^1^ and provide trillions of dollars of ecosystem services each year such as coastal protection, fisheries, and tourism^2^. Cold-water corals found in the deep sea also form reef structures and are important ecosystem engineers, underpinning deep water marine biodiversity in many regions^3^. All stony corals make their typically aragonite skeletons from Ca^2+^ and CO_3_^2−^ dissolved in seawater. However, the precise processes involved are unclear, thus limiting the accuracy in projections of the impacts of future climate change on these important ecosystems and undermining the accuracy of palaeoclimate reconstructions based on skeletal elemental and isotopic compositions. Collectively such biomineralisation processes are known as “vital effects”; a catch-all term for biological processes that may complicate proxy-based environmental signals preserved in a coral skeleton^4^.

The degree to which coral biomineralisation is a biological versus physiochemically controlled process is hotly debated^5^. Models for both pathways agree that calcification occurs in a privileged space^6^, and that the organism has overall control on the calcification process, but they disagree in terms of what processes control the initiation and subsequent growth of the skeleton. The biological model favours crystal growth by “particle attachment” of intracellularly grown amorphous calcium carbonate (ACC) that gradually crystallise to aragonite^7^. Whereas the physiochemical model suggests crystal growth is achieved via more classical “ion attachment” processes in a calcifying fluid supersaturated with respect to aragonite^8^. Sun et al.^9^ propose an alternative that incorporates aspects of both previous models—ACC particles are grown intracellularly in vesicles of the calcifying fluid and attached to the growing crystal where they crystallise. The space between ACC particles is then filled by growth of aragonite crystals principally via ion attachment. While this combination model explains a number of recent observations on the occurrence and distribution of ACC in freshly grown coral skeleton, the presence of an ACC precursor phase is not universally accepted^10^ and the model’s implications for skeletal geochemistry are currently unknown.

In contrast to their debated biomineralisation mechanisms, the morphology and crystal structure of coral skeletons is well described and understood^11^. Skeletal extension is accomplished through the formation of the organic-^12^ and Mg-rich^13^ centres of calcification (COCs^14^). Crystals within COCs are randomly orientated and act as the scaffold for successive growth of radially orientated elongated acicular low-organic and low-Mg crystals, termed fibres, that thicken the skeleton. Although COCs in mature skeletons are thought to contain ACC^7,9^, and appear at a micron-scale to be more granular than the fibres, at a nanoscale both microstructural elements show clear evidence of growth via particle attachment and ion attachment^7,9^. Previous studies have documented a suite of geochemical differences between these structural elements. For instance, elevated Li/Ca and Mg/Ca in the COCs compared to surrounding fasciculi (the fibrous crystals) has been documented previously in both deep-sea and tropical coral species, including *D. dianthus*, *Lophelia pertusa*, *Porites clavus*, and *Porites lobata*^13,15–20^; elevated Ba/Ca has also been reported in the COCs of some tropical corals compared to the fasciculi^15^; Sr/Ca is typically, but not always, enriched in COCs^16,19,21–24^; whilst B/Ca, U/Ca, δ^13^C, and δ^11^B, are typically lower within the COCs compared to the fasciculi^19,20,25–29^. Adkins et al.^28^ also documented an additional δ^18^O depletion in COCs compared to the δ^13^C-δ^18^O trends defined by the fibres, although subsequent studies seem to have failed to replicate this finding^20^.

Deep-sea corals are highly suited to the exploration of vital effects because, compared to the high diurnal and seasonal cyclicity in tropical shallow water reef corals, they experience relatively stable environment during life and are azooxanthellate and are thus free from potential microenvironmental changes caused by symbionts^3,29–31^. In species such as *D. dianthus*, the skeletal micro-structural components tend to be relatively large, with COCs (cross sectional COC thickness of ~ 100 μm) occurring less frequently than in many tropical coral species where they are also smaller (cross sectional COC thickness of ~ 10 μm). As a result, variable proportions of COCs to fibres in bulk samples of deep-sea corals have been invoked to cause scatter in bulk geochemical measurements, thereby contributing to vital effects and increasing the uncertainty in climate proxy studies^19,32^. Because of the small size of the COCs relative to the size of a typical sample, this is not thought to be an issue for sampling tropical corals. However, Sinclair^33^ and Sinclair et al.^25^ noted that the seasonal cycles of Sr/Ca, Mg/Ca, and U/Ca in the skeleton of the tropical coral *Porites* could also be largely caused by seasonally variable oscillations in the relative contributions of COC and fibres to the bulk sample. While thermodynamic considerations and other effects (e.g. Rayleigh fractionation, precipitation rate) have also been proposed to explain seasonal cycles in element to calcium ratios (E/Ca) in tropical corals^18,21,25^, a greater understanding of the causes of geochemical variation between these two structural components is clearly desirable to better understand the controls on coral-based climate proxies, and to identify the relative importance of inorganic vs. biological drivers of the variations that form the basis of the proxies.

**Figure 1:**
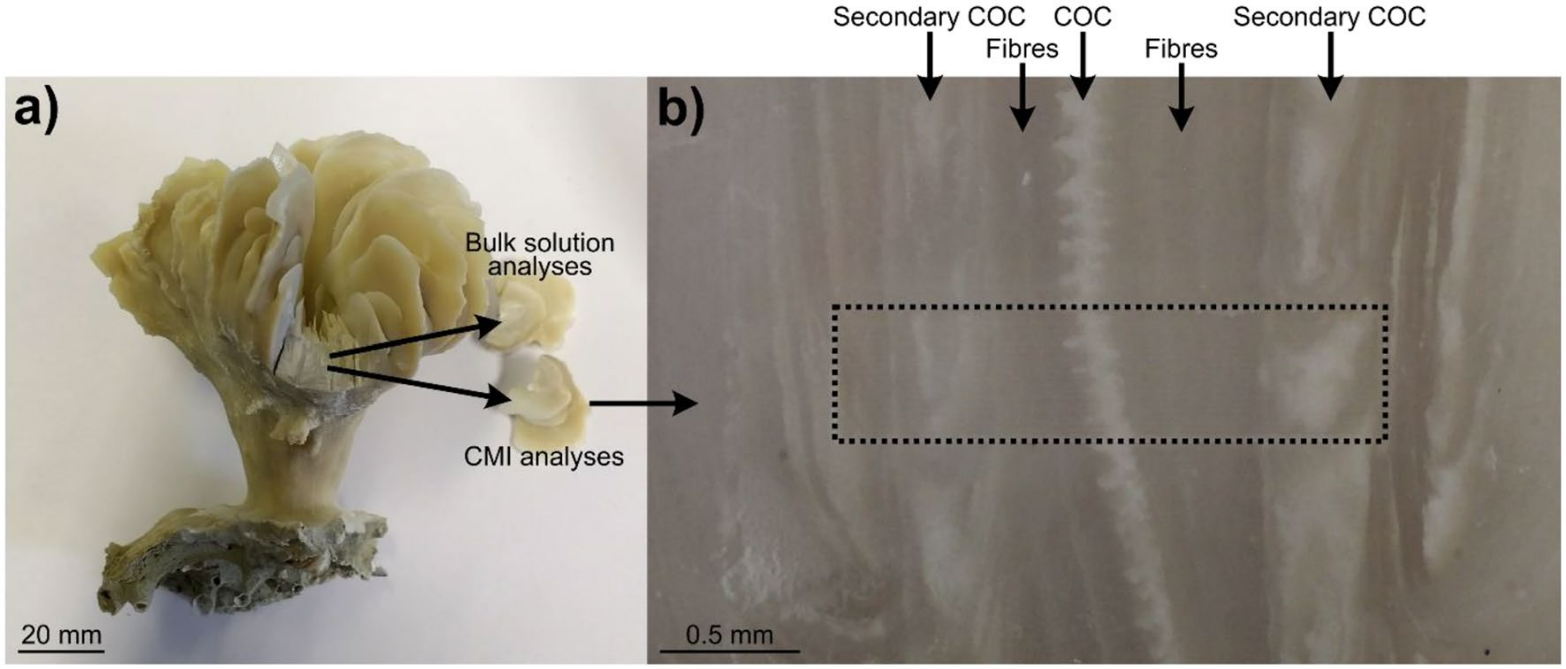
*D. dianthus* DY081-914DD: (**a**) removal of septa from specimen for both bulk solution geochemistry (Stewart et al*.*^41^; Kershaw et al.^42^; this study) and CMI; (**b**) optical image of the surface area of DY081-914DD where CMI was performed (approximated by dashed box). The primary COC is visible running vertically down the centre of the optical image as a white band, surrounded by dark fibrous aragonite on each side. Secondary COCs consist of a mixture of COC-like white and fibre-like dark material.

A rapidly growing area in the Life Sciences is the concept of correlative multimodal imaging (CMI), where images from a diverse range of techniques (modalities) are precisely combined to create a composite and holistic view of the structure, function, and composition, of a sample from cm to sub-μm scales. So much so, CMI now underpins many novel and emerging approaches in the Life Sciences^34,35^, yet CMI approaches tend to be under-utilised in the Earth and Ocean Sciences despite the imaging and in situ analytical techniques used in many approaches at the forefront of the field^36–39^. Here, for the first time, we employ a CMI approach to combine optical, 2D elemental (Li/Ca, B/Ca, Mg/Ca, Sr/Ca, Ba/Ca, U/Ca), isotopic (δ^11^B), and Coherent Anti-Stokes Raman Scattering (CARS) Microscopy (targeting organic distribution), to study a sample of *D. dianthus* (sample ID: DY081-036-ROV335-Ev052-202-914DD-Lrg; here after DY081-914DD; Fig. 1) collected in 2017 from the Labrador Sea (RRS Discovery DY081) at a latitude of 63.33° N, longitude of − 52.77° E, and depth of 1137 m (hydrographic data are presented in Supplementary Table S1). The sample encompasses both a primary COC (i.e. clearly visible and well defined COCs), secondary COCs (fainter and less well defined COCs), and fibrous aragonite which when imaged allow us to fully document the spatial variation in skeletal composition and unambiguously isolate the composition related to each structural component. Obtaining geochemical information isolating just a single component has proven very difficult in previous studies (e.g. by micro-milling powders^19,20^), but by combining this with established approaches using the boron-based tracers of calcifying fluid composition (pH and [CO_3_^2−^]^40^; Materials and Methods), we provide novel insights into the mechanisms of biomineralisation in the skeleton of the stony deep-sea coral *Desmophyllum dianthus*.

## Results

**Figure 2:**
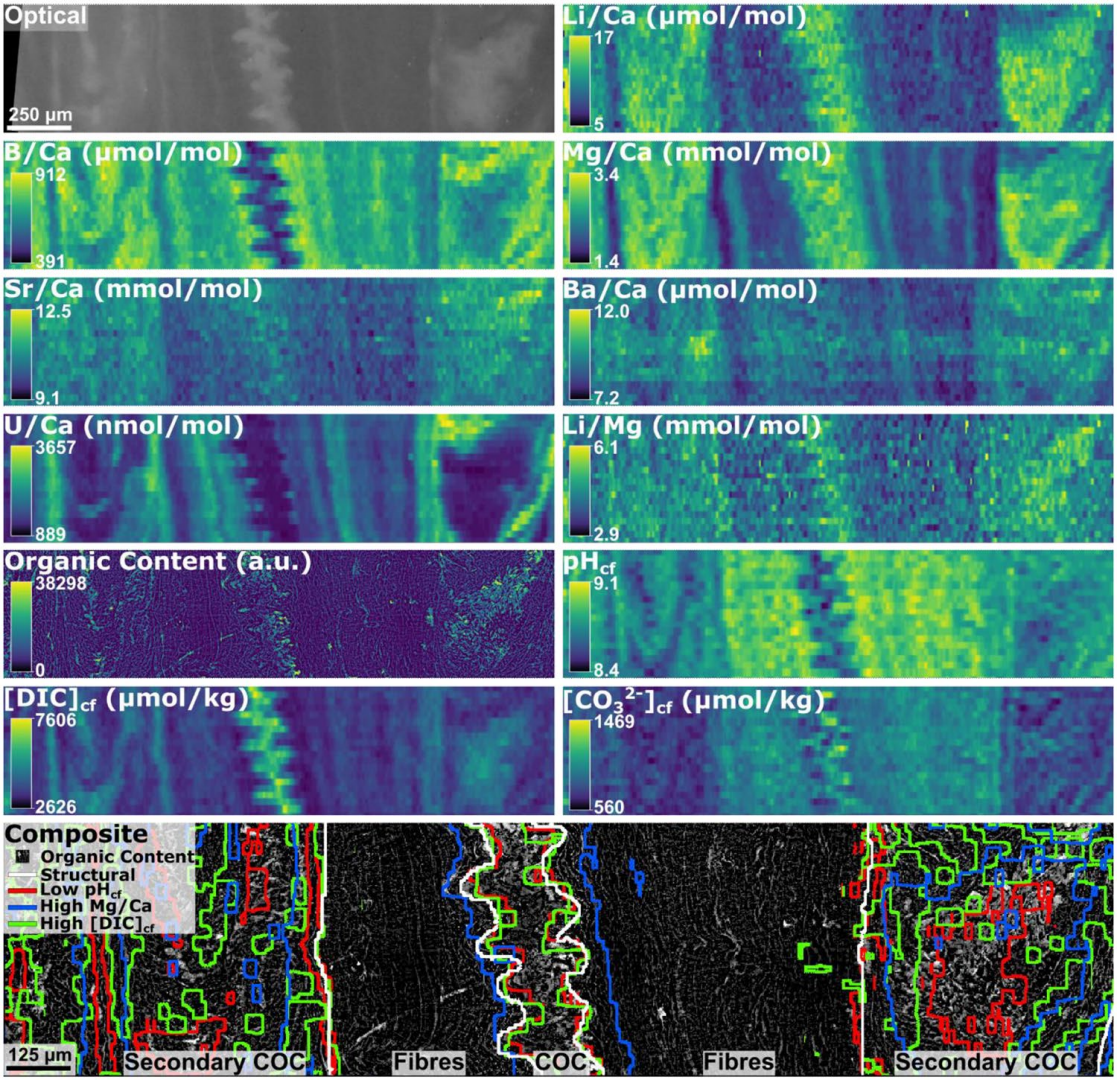
Optical and geochemical imaging of DY081-914DD collected on a range of modalities, along with an aligned, composite image where a greyscale version of the organic content image is overlain by lines identifying locations of low pH (red), high Mg/Ca (blue), and high DIC (green) (where low and high constitutes the lower and upper 50% of values respectively). Structural component boundaries identified using the optical image are shown by bold white lines.

Laser ablation inductively coupled plasma mass spectrometry (LA-ICP-MS) data were used to create a series of 2D geochemical images, and alongside the CARS image of CH bond intensity, this results in a series of cross-sectional views through the skeleton of *D. dianthus* DY081-914DD for a range of geochemical variables encompassing the dominant microstructural variation (Fig. 2, Supplementary Video S1, Supplementary Data File).

The mean (± 1 SD) elemental and isotopic compositions of the area of *D. dianthus* DY081-914DD imaged are as follows: Li/Ca = 10.68 ± 2.70 µmol/mol, B/Ca = 706.28 ± 79.74 µmol/mol, Mg/Ca = 2.40 ± 0.49 mmol/mol, Sr/Ca = 10.27 ± 0.40 mmol/mol, Ba/Ca = 9.05 ± 0.68 µmol/mol, U/Ca = 1715.63 ± 497.63 nmol/mol, Li/Mg = 4.40 ± 0.47 mmol/mol, δ^11^B = 25.80 ± 1.71 ‰ (Supplementary Table S3). These values are consistent with bulk samples of skeleton from the same coral analysed by solution ICP-MS both here and elsewhere^41,42^ (Supplementary Table S2, Supplementary Fig. S1); mean LA values are within 11% of bulk samples for all geochemical parameters. Mean (± 1 SD) carbonate system parameters for the calcifying fluid, calculated from the B/Ca and δ^11^B (see Materials and Methods), are as follows: pH_cf_ = 8.78 ± 0.12, [DIC]_cf_ = 3802.8 ± 666.0 µmol/kg, [CO_3_^2−^]_cf_ = 883.1 ± 132.2 µmol/kg, and saturation state (Ω_cf_) = 11.42 ± 1.71 (Supplementary Table S3).

**Figure 3:**
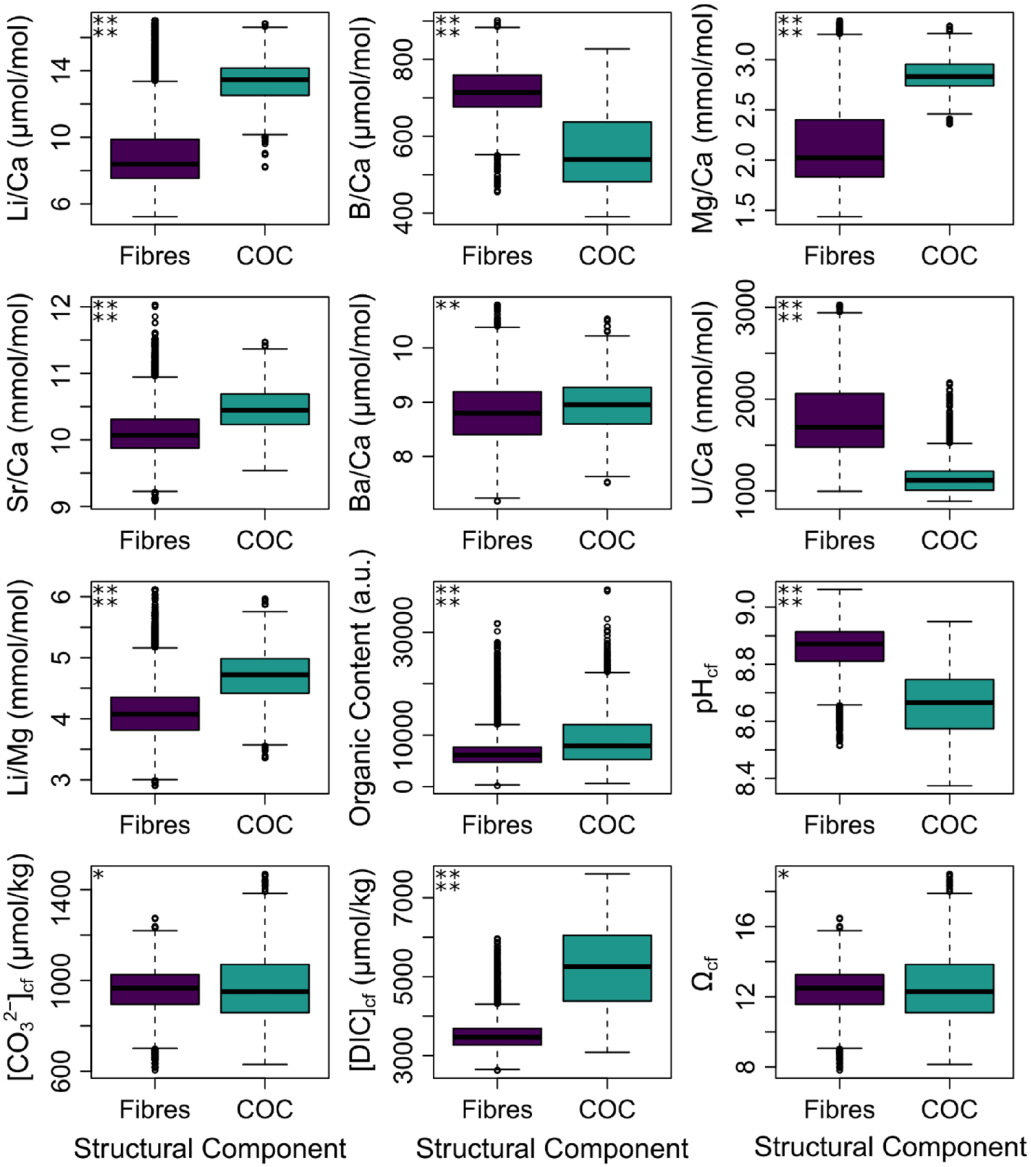
Boxplots showing the geochemical composition of the primary COC and fibrous aragonite of DY081-914DD. Results of size effect analysis (Cohen’s d) are shown in the top left corner of each panel: four stars = large d-value, three stars = medium d-value, two stars = small d-value, and one star = negligible d-value.

CMI can be performed between the optical image and the geochemical data collected on a range of instrumentation, permitting direct comparisons between the visible micro-structure and the geochemical parameters studied (Fig. 2). Manual segmentation of the optical image to highlight the three key structural components—the primary COC, secondary COCs and fibrous aragonite—demonstrates that considerable spatial variations in the composition of the coral skeleton are associated with the microstructural components of the coral: for example the central COC is characterised by higher organic content and higher Li/Ca, Mg/Ca, Li/Mg, Sr/Ca, Ba/Ca, [DIC]_cf_, [CO_3_^2−^]_cf_, and Ω_cf_, and lower B/Ca, U/Ca, δ^11^B and pH_cf_, compared to the fibrous aragonite (Supplementary Table S3). Following correlative analysis, the composition of the primary COC, the secondary COCs, and the fibrous aragonite can be extracted from the images (see Materials and Methods; Fig. 3). The secondary COCs appear in the optical image as a complex mix of COC-like and fibre-like aragonite, consequently hereafter we focus on the difference between the primary COC and the fibrous aragonite only. Effect Size analysis (Supplementary Table S4) was performed to assess the degree of difference between the COC and fibrous aragonite means (Cohen’s d). Differences are large for all geochemical parameters (at 95% confidence) except calculated [CO_3_^2−^]_cf_ and Ω_cf_ (d-value 0.11), and measured Ba/Ca (d-value 0.28).

## Discussion

**Figure 4:**
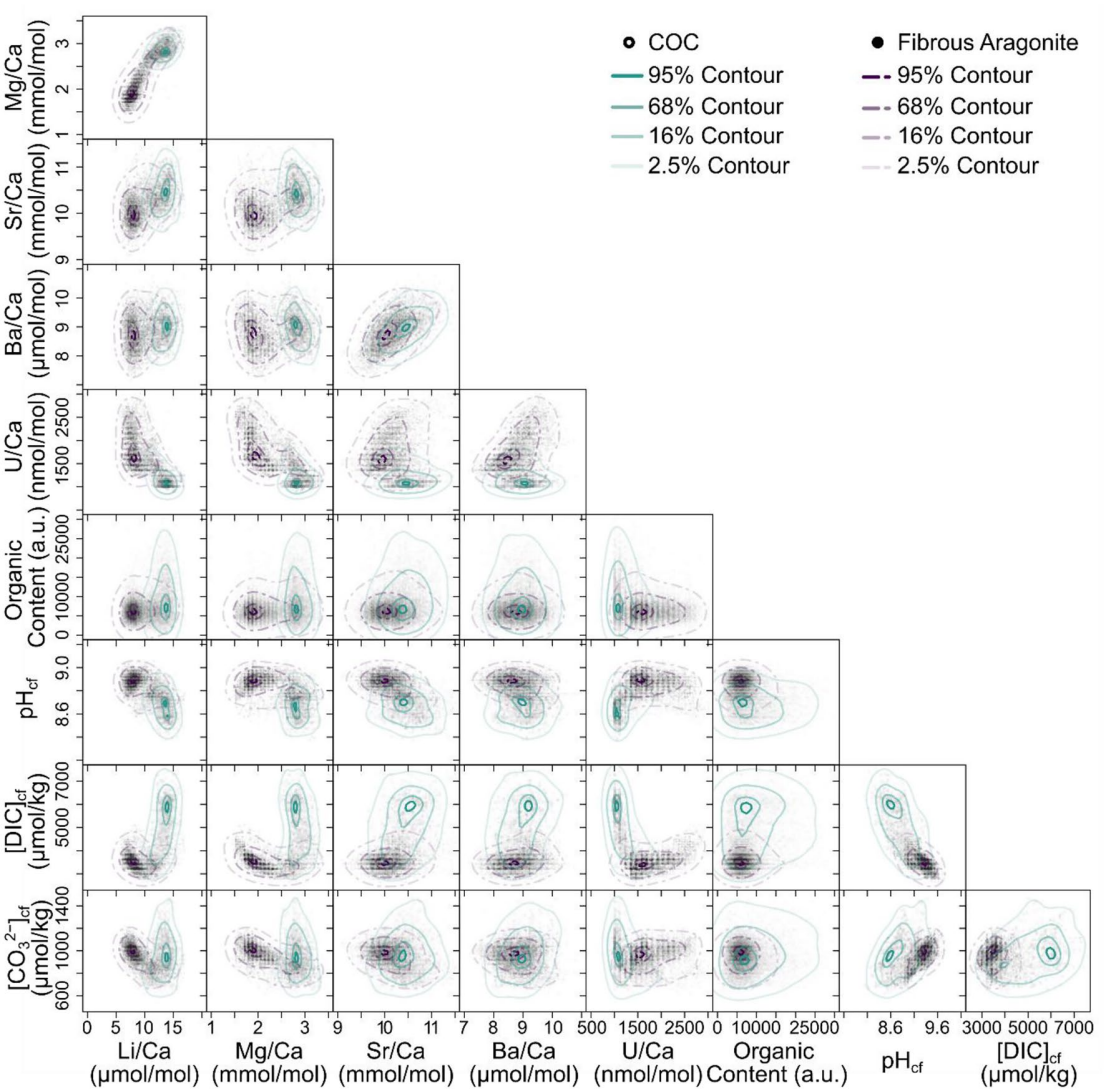
Cross plots of selected geochemical variables for DY081-914DD. All pixel data are plotted as semi-transparent black data points to give a sense of data density. Data for the COC and fibrous aragonite are contoured at confidence levels of 95%, 68%, 16%, and 2.5% (solid turquoise and dashed purple lines), respectively.

The key finding of our novel CMI approach is that the geochemical differences recorded between the fasciculi and COC of *D. dianthus* DY081-914DD are best explained by invoking contrasting biomineralisation mechanisms. The geochemical variations recorded are, for the most part, consistent with the results of previous studies, with the exception that with our CMI approach we are able document a clear distinction between micro-structural components (Fig. 4). For instance, we see elevated Li/Ca and Mg/Ca in the COCs compared to surrounding fasciculi^13,15–20^ (see Supplementary Figure S2 for a comparison to data from Chen et al.^20^). In contrast to Shirai et al.^24^ and Gagnon et al.^16^, but similar to others^21–23^, we see Sr/Ca is elevated in the COC, while B/Ca, U/Ca, and δ^11^B are lower compared to the fasciculi, consistent with numerous studies^25–27,29,39^ but not Stewart et al.^19^. Where differences exist, they can likely be ascribed to either differences between coral species or the different sampling strategies employed, as obtaining material exclusively from COCs is highly challenging through the frequently used micromilling approach. Our CARS image documents the location of organics and clearly these are elevated in the COC (Figs. 2,3, and 4) as noted elsewhere^12,43^.

**Figure 5:**
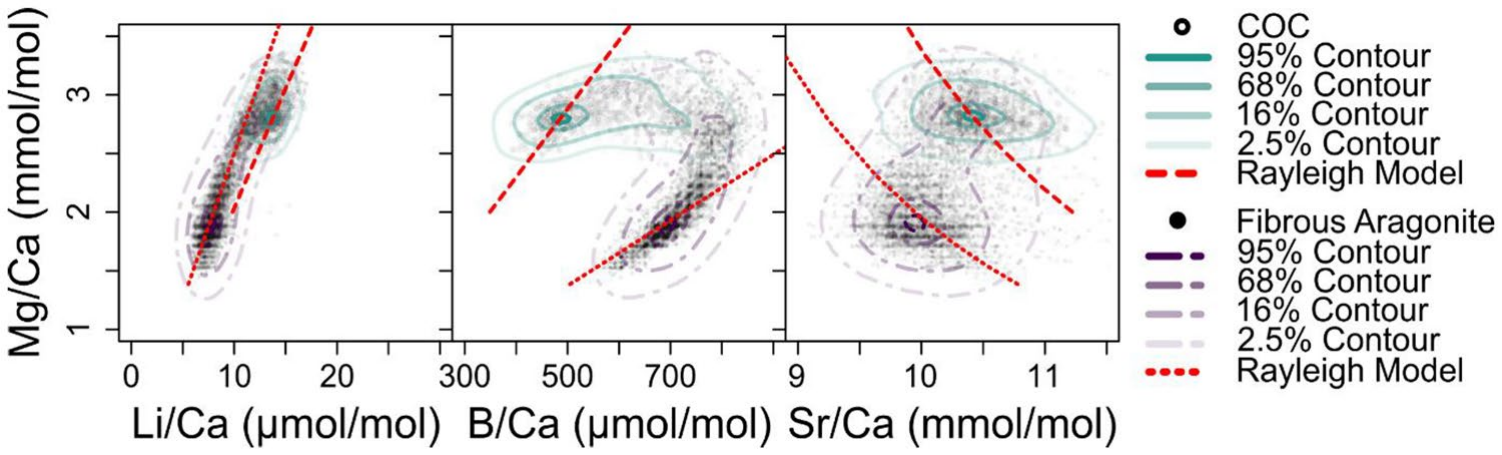
Rayleigh fractionation models (red dashed line) for Li, B, Mg and Sr incorporation into the aragonitic skeleton of DY081-914DD. All pixel data are plotted as semi-transparent black data points. Data for the COC and fibrous aragonite are contoured at confidence levels of 95%, 68%, 16%, and 2.5% (solid turquoise and dashed purple lines), respectively.

Based on the patterns observed between the elemental ratios, it does not appear that all the elemental variability is simply a result of two component mixing^33^. Although for some elements such mixing could explain the observed trends (e.g. Mg/Ca vs. Li/Ca; Fig. 4), in others the lack of a linear relationship means it cannot (e.g. U/Ca vs B/Ca; Supplementary Fig. S2). Furthermore, while the elemental data for the primary COC often forms more of a cluster, for the fibrous component the data tend to form an array (e.g. Mg/Ca vs. Li/Ca, Mg/Ca vs. B/Ca; Fig. 5). Consistent with several previous studies^16,20^, a simple closed system Rayleigh-type fractionation model for the incorporation of elements into the skeletal aragonite (see Materials and Methods) can broadly replicate the arrays seen in the fibrous aragonite data, suggesting Rayleigh fractionation has an important role to play in causing compositional variation within the fibrous components of coral skeletons. Rayleigh models do not replicate the COC data as well, suggesting such fractionation has less of a role in the compositional variation within the COC components; a conclusion that aligns with previous work^16^. Regardless, it is clear that Rayleigh fractionation cannot explain the compositional variation between the fasciculi and COCs (Fig. 5 and Supplementary Figure S3).

Previous studies have shown boron-based tracers of calcifying fluid carbonate chemistry to be in good agreement with independent pH measures^44^. Although widely applied^40,44,45^, recent studies have highlighted the complexity of comparing direct and indirect measures of carbonate chemistry in corals^46^ and raised questions about the veracity of B/Ca as a tracer of fluid DIC^47^. Nonetheless, taking the boron systematics at face value, we find the pH of the calcifying fluid to be lower, and the [DIC] to be higher, in the COCs compared to the fasciculi (Figs. 2,3, and 4). Furthermore, despite lower pH_cf_, [CO_3_^2−^]_cf_ (and hence Ω_cf_, assuming unmodified seawater [Ca^2+^]) is similar in both the primary COC and fasciculi. There is no correlation between pH_cf_ (calculated using δ^11^B and Eq. 1) and Mg/Ca (Fig. 4) which suggests Rayleigh fractionation does not dominate the boron isotopic systematics in the fibrous component from which the pH_cf_ is derived (consistent with the findings of Stewart et al.^19^). But, given the B/Ca and Mg/Ca co-variation, notably in the fibres, (Fig. 5), trends in [DIC]_cf_ and the other carbonate system parameters calculated from B/Ca should be interpreted with caution. Support for such contrasting carbonate systematics of the COCs and fibres comes from U/Ca, a tracer inversely related to [CO_3_^2−^]_cf_^48^, that is also clearly distinct between the structural components (Figs. 3 and 4).

**Figure 6:**
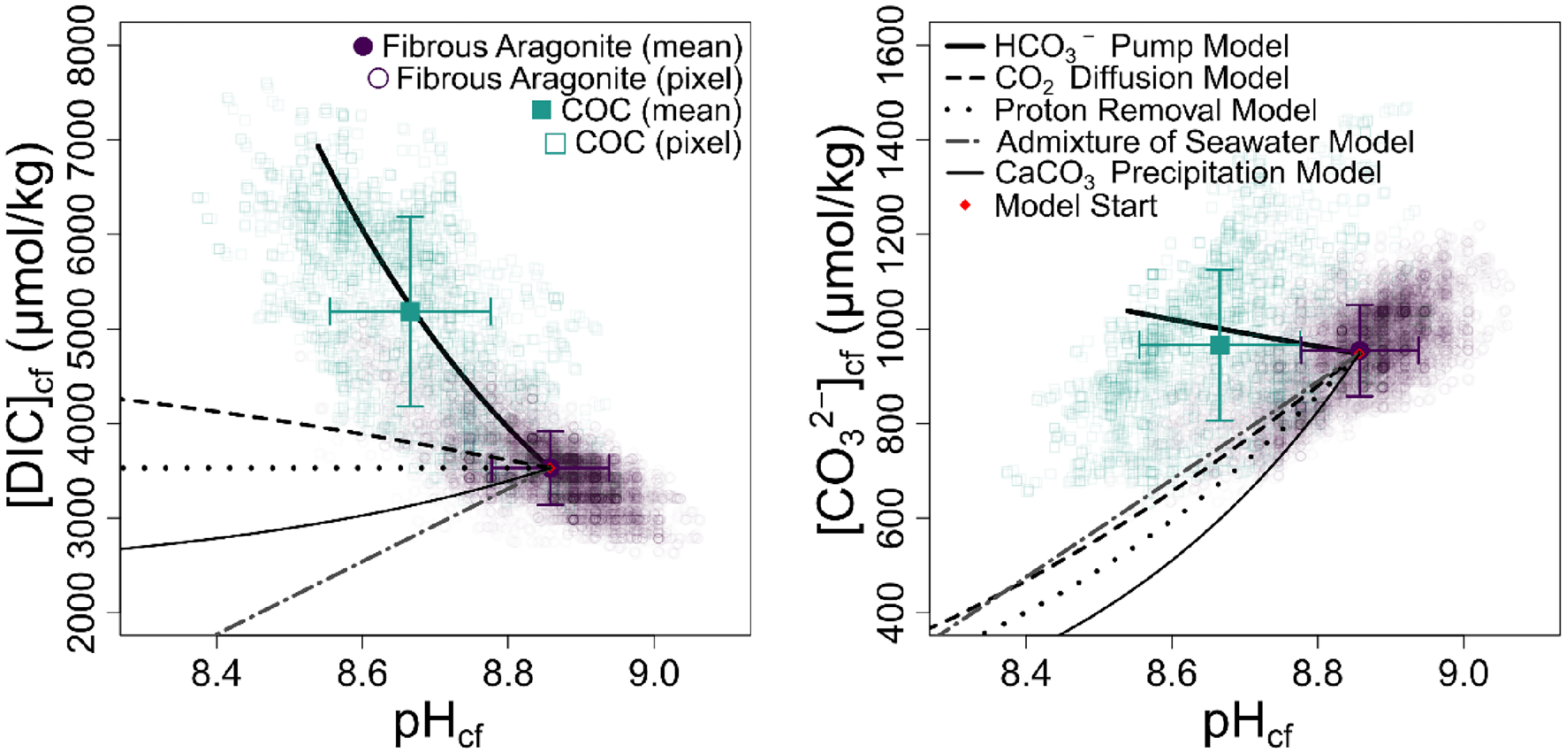
[CO_3_^2–^]_cf_ and [DIC]_cf_ versus pH of DY081-914DD. The trend lines show models for the evolution of the carbonate fluid as: (i) HCO_3_^–^ is added, simulated by increasing [DIC]_cf_ and ALK in a 1:1 ratio, (ii) metabolic CO_2_ diffuses in, increasing [DIC]_cf_ with alkalinity remaining constant, (iii) a reduction in the removal of protons by the CaATPase enzymatic pump, decreasing alkalinity with [DIC]_cf_ remaining constant, (iv) admixture of seawater into the calcifying space, and (v) precipitation of CaCO_3_ that decreases alkalinity and [DIC]_cf_ in a 2:1 ratio. Regarding COC and fibrous aragonite, filled symbols denote mean compositions (± 1 SD) whilst open symbols are that of individual pixels.

Guo^49^ presented a model whereby the state of the carbonate system in the calcifying fluid is determined by the balance between: (1) the degree of proton removal by the Ca-ATPase enzymatic pump which increases pH_cf_ and does not directly influence fluid [DIC]_cf_; (2) the degree of [DIC]-enrichment caused by either invasion of diffused metabolic CO_2_ or the active pumping of HCO_3_^−^ by bicarbonate ion transporters, both of which cause pH_cf_ to decline and calcification fluid [DIC] to increase; (3) precipitation of CaCO_3_ that, by removing 2 M of alkalinity and 1 M of [DIC] per 1 M of CaCO_3_, lowers both [DIC]_cf_ and pH_cf_; and (4) the admixture of seawater into the calcifying space that partially (or totally) returns the internal carbonate system back towards external seawater values. Applying a simplified version of the model, with no feedback between Ω_cf_ and CaCO_3_ precipitation (Fig. 6), suggests that the [DIC]_cf_ and pH_cf_ differences recorded here between the COCs and fibres of DY081-914DD can primarily be explained by greater HCO_3_^−^-enrichment within the fluid from which the COCs formed, either through active pumping of HCO_3_^−^ by bicarbonate ion transporters or via a greater diffusion of CO_2_ that is converted to HCO_3_^–^ and H^+^ via carbonic anhydrase and the H + pumped away^50^. This contrasts with similar work by Fietzke and Wall^39^ who concluded that simple pH_cf_-DIC_cf_ up-regulation could not explain the carbonate chemistry of the COCs of *L. pertusa*. Their isotope data implies a much lower pH_cf_ or an additional source of borate ion, with active transport of seawater borate to the COCs by bicarbonate anion transporters proposed as a possible mechanism. We see no need to invoke such processes here, and future studies should explore whether species-specific differences in calcification mechanisms exist amongst both deep-sea and tropical corals.

Recent models of calcium carbonate biomineralisation in both marine calcifiers generally, and corals specifically, have favoured an ACC precursor phase^7^. It is thought that this phase forms in vesicles of intracellular calcifying fluid, and that the resulting nanoparticles of ACC-H_2_O are subsequently exocytosed into the extracellular calcifying fluid where they attach to the biomineral growth front (particle attachment). Further biomineral growth via ion attachment follows in the extracellular calcifying fluid^6^, which fills the interstitial space between the attached particles^6,9^. It has previously been recognised that Mg ions are an important component of ACC with a role in its formation and stability, and the same is the case for organic compounds^51–54^. Nanoscale particles of ACC have been observed by Sun et al.^9^ at or near the forming surface of coral skeletons in a number of species. Whilst not identified in the mature and fully formed skeletal fibres, they were found to persist to some degree in the COCs^7,55,56^. We therefore propose that the COC in DY081-914DD, enriched in Mg and organics (Figs. 2,3, and 4), has a geochemical signature more influenced by a non-classical crystallisation pathway, likely using particle attachment of an ACC precursor. By contrast, we propose that a different biomineralisation mechanism is responsible for the formation of the fibrous aragonite, and that this is less reliant on organics. One possibility is that it involves a greater role for the classical crystallisation pathway of ion attachment, because ion-by-ion crystallisation from a semi-enclosed reservoir is more consistent with the Rayleigh fractionation we observe in these components. This does not mean we rule out some degree of Rayleigh fractionation in the formation of COCs as well, but given the less well-defined arrays in Fig. 2 we consider this an open question. The boron systematics lend further support to the above since the model of Gilbert et al.^6^ conceptualises an intracellular calcifying fluid originating initially from the extracellular calcifying fluid being modified by an additional flux of CO_2_ and/or HCO_3_^−^ due to the higher surface area of the small vesicles^6,9^, adequately explaining the DIC-enrichment (and corresponding pH decline) we observe in that component.

A notable feature of our reconstruction of the calcifying fluid pH is that although the [DIC]_cf_ is clearly enriched in the fluid that deposited the COC, the [CO_3_^2-^]_cf_ is only slightly higher than for the fluid that deposited the fibres. At face value this could support a greater role for a biological control on biomineralisation in the COC via an ACC precursor, where nucleation is controlled more by the secretion of organic molecules (e.g. Coral-Acid Rich Proteins^7^) rather than a physiochemical increase in saturation state (the opposite may be true for the fibres). However, given we do not know the [Ca] of the calcifying fluid it is possible that the aragonite saturation state of the fluids depositing the COC and fibres were more divergent than they appear in Figs. 3 and 4. We should also caveat that this discussion on boron isotope systematics ignores the potential influence of boric acid diffusion^57^ and, if we are correct that COC formation involves a significant ACC component, then we may not be able to apply the boron system in the straightforward way we have done here, since the use of B/Ca to provide the second carbonate system constraint is based on aragonite grown using classical crystallisation methods^43^. The available literature is currently limited, and while there is no clear fractionation between aragonite grown via a classical crystallisation pathway and ACC in terms of δ^11^B^58,59^, isotopic fractionation can be induced by the process by which ACC is subsequently converted to aragonite, which depends on experimental conditions and set up ^57^. Notably, the partition coefficient of B/Ca into ACC is up to 4 orders of magnitude higher than for abiogenic aragonite^58,60,61^. This contradicts our observation that B/Ca is lower in the COC than fibre, which either supports the notion that an ACC precursor is not a significant phase of coral biomineralisation^10^ or highlights that more work is needed to better understand the consequences of including non-classical crystallisation pathways in our models of proxy systems.

The hypothesis that different mineralisation strategies underpin the formation of COCs and fibrous aragonite in *D. dianthus*, and that these differing processes drive the geochemical variations recorded between these two key skeletal components, is likely also the case for other species of both cold-water and tropical coral. After all, similar geochemical variations have previously been recorded in a host of species^15–27^. Here we favour variations in the importance of particle attachment and ion attachment as the key driver behind these differences, but there is no reason why this must be the case for all species of coral. Further investigations are clearly required.

Our CMI approach has enabled us to cleanly sample both the COC and fibrous components of *D. dianthus*, and to precisely combine E/Ca, boron isotopes, carbonate chemistry, and organic content, to give a highly spatially resolved (µm-scale) view of the coral’s skeletal geochemistry. Previous studies concerned with the environmental signals preserved in coral skeletons typically do not target any particular microstructural component, rather relying on bulk sampling to average out any variability that might arise from preferentially sampling COC or fibrous aragonite (*cf*. Ref. 19). In this context “vital effect” is a catch-all term for any and all biological processes that may complicate proxy-based environmental signals preserved in a coral skeleton^4^, and we further hypothesise that a currently unknown proportion of a coral’s vital effects can be explained by variable mixing of the geochemically distinct COC and fibre components, supporting an idea that has been proposed previously^25,33^.

The implication is that a coral’s package of biomineralisation strategies is itself a vital effect. With respect to *D. dianthus* we argue that this can be considered a key vital effect because the coral’s relatively large COCs (~ 100 µm thick) provide ample material for mixing with fibrous aragonite during sampling. Other species, including those living in tropical waters, can be characterised by much narrower COCs on the order of 10 µm thick, thus the impact on coral geochemistry of mixing COC and fibrous components may be lessened. However, in such taxa COCs are more frequent and fibrous regions are less voluminous compared to *D. dianthus*. Given that it is the ratio of COC to total skeleton that will determine the impact of COC-fibre mixing for a given species, the impact is likely to be still noticeable, as found by Sinclair^33^ and Sinclair et al.^25^. An important avenue of future research is therefore the quantification of COC:total skeleton for a range of species if we are to fully understand the impact of biomineralisation strategy on coral geochemistry more widely.

## Conclusions

Our correlative imaging approach can unambiguously isolate the geochemical signatures associated with each micro-structural component of the skeleton of *D. dianthus*. This treatment highlights that it is likely that the biomineralisation toolkit applied by *D. dianthus* varies across the skeleton between micro-structural elements, compounding to generate the spatially variable geochemistry clearly observable in our 2D images (Fig. 2; Supplementary Video S1), and contributing to the array of vital effects that obscure environmental records preserved in the geochemistry of coral skeletons. Indeed, the ability of a CMI approach to cleanly isolate the signature of the fibrous aragonite component means that it can be used to unpick coral vital effects, thus revealing a wealth of previously obscured geochemical information and higher fidelity proxy data reflective of the environmental conditions in which the corals mineralised^19^.

Our proposal for a stronger biological influence on the formation of COCs with a greater involvement of ACC calls for further study into the geochemical characteristics of ACC and its subsequent transformation into aragonite. Unless it can be established that the proportion of COC is relatively stable, following previous work^19^ we recommend studies focusing on reconstructing past climates target the fibrous aragonite portion of coral skeletons as these may have formed by ion attachment (although ACC could still play a more subordinate role), a process more analogous to the inorganic experiments that underpin the current understanding of many proxies^62^ and less likely to be affected by physiological changes.

Given that coral skeletons extend through the deposition of COCs, with the fibres subsequently thickening the skeleton^14^, different environmental sensitivities of the spatially variable biomineralisation toolkit employed by corals such as *D. dianthus* may ultimately underpin the response of the phenotype to environmental change^63^. There is a pressing need to gain a mechanistic understanding of coral calcification and its environmental sensitivity to better predict and comprehend their response to anthropogenic global climate change.

## Materials and methods

### Sample preparation

Following collection, the sample was air dried and coral tissue was removed by physically scraping the specimen then treating it in dilute NaClO for 12 h. Whole “S1” septa and attached theca were taken from the sample. For CMI, one such septa was sectioned using a high carbon sintered diamond rotating saw and de-contaminated using silicon carbide (SiC) fixed abrasive P1200 grit papers to remove any metal residue left from the blade. The sectioned piece was mounted in MetPrep EpoFLO high purity, low viscosity clear epoxy resin, then the mounted sample was ground using a high concentration (HC) sintered diamond rotating grinder to expose the coral surface. Once exposed, the sample was ground using fixed abrasive P1200 SiC grit paper to prepare it for polishing. This was performed firstly using Kemet PSU-M polishing cloths (15 µm, 9 µm, and 3 µm grades) with diamond in oil suspension, before a final polish was carried out using Al_2_O_3_ 0.3 µm with water. After polishing, the sample was cleaned by ultrasonication in alcohol followed by ultrapure water for 5 min. The sample was optically imaged using a Canon EOS 60D digital camera connected to an Olympus BX60 microscope.

### Bulk δ^11^B and elemental analysis

We compare our high spatial resolution dataset to geochemical analyses of the bulk DY081-914DD carbonate skeleton. Many of these results have been published previously including Li/Ca, Mg/Ca, Ba/Ca, and Sr/Ca ratios^41,42^. Additional bulk Fe/Ca and Mn/Ca values recorded in this specimen suggest that the cut samples were are largely free from Fe-Mn coating contamination (both < 0.15 µmol/mol^42^). To supplement this, we present measurements of bulk B/Ca, U/Ca, and δ^11^B performed on the same sample replicates (oxidatively cleaned) used in Stewart et al.^41^. All bulk data from this study and previous publications are given the supplementary information (Supplementary Table S2).

An aliquot of the dissolved sample was analysed using well-characterised, matrix-matched, synthetic standard solutions to yield B/Ca and U/Ca ratios using a Thermo Element2 ICP mass spectrometer at the University of Bristol. Repeat analysis of NIST RM 8301c (n = 35) and JCp-1 (uncleaned) carbonate reference materials yielded analytical precision of <  ± 2% (1σ) for these elemental ratios and average values within 1% of interlaboratory consensus values^64,65^.

For bulk δ^11^B analysis, boron was separated from the carbonate matrix using Amberlite IRA 743 micro-columns following the protocol of Foster et al.^66^. δ^11^B was then measured by multi-collector (MC)-ICP mass spectrometer against NIST SRM 951 at the University of Bristol. Samples, blanks, and standards were introduced to the instrument in a 0.5 M HNO_3_ and 0.3 M HF acid matrix to aid B wash out. Full procedural uncertainty was assessed using repeat measurements of NIST RM 8301 (Coral) consistency standard^65^ yielding an average δ^11^B value of 24.24 ± 0.15‰ (2SD; n = 67).

### Boron isotope analysis by LA-MC-ICP-MS

Boron isotope analyses were performed on a Thermo Scientific (Thermo Fisher Scientific, Waltham, MA, USA) Neptune Plus MC-ICP mass spectrometer coupled to an Elemental Scientific Lasers (Bozeman, MT, USA) NWR193 excimer laser ablation system with a TwoVol2 ablation chamber. Analytical protocols broadly followed Chalk et al*.*^36^. ^10^B and ^11^B were measured on the L3 and H3 Faraday cups installed with 10^13^ Ω resistors. Potential surface contamination was removed prior to data collection by ablating the sample and standard surfaces with a low laser power density (~ 1.5 J cm^−2^) and fast laser tracking speed and repetition rate (200 μm s^−1^ and 20 Hz, respectively). Operating conditions during data acquisition are detailed in Supplementary Table S5. Data were collected in static mode, with each analysis consisting of 192 integration cycles of 1.051 s. Samples and standards were ablated in line-mode, with each ablation line being ca. 2 mm in length. The sample analyses consisted of 20 parallel and adjacent ablation transects with a laser beam size of 25 μm^2^, resulting in an ablated area ca. 0.5 by 2 mm. Dynamic blank corrections were applied cycle by cycle assuming a linear relationship between the preceding and succeeding blank measurements, and instrumental mass bias was corrected by standard-sample bracketing with NIST SRM610 glass reference material and the isotope composition published by Standish et al*.*^67^. A matrix interference from scattered Ca ions on the boron mass range^67^ was corrected using the relationship between δ^11^B inaccuracy and ^11^B/Ca_interference_ (following the power law^68^) of carbonate reference materials JCp-1, *Porites sp.* coral and JCt-1, *Tridacna gigas* clam^69,70^. Standard and sample data were screened and cycles falling outside of 2 SD (standards) or 4 SD (samples) of the mean were removed. Internal reference material PS69/318-1, a fragment of cold-water coral, was ablated throughout the analytical session as a guide to internal precision, external reproducibility, and accuracy.

Internal precision, expressed as 2 SE of the mean of the 192 integration cycles, was ≤ 0.6‰. The mean δ^11^B of the repeat analysis (n = 5) was 13.77 ± 0.43‰ (2 SD), consistent with a solution measurement of 13.83 ± 0.29‰ (2σ)^67^.

### Elemental analysis by LA-ICP-MS

Elemental analyses were performed on an Agilent (Agilent Technologies Inc., CA, USA) 8900 Triple Quadrupole ICP mass spectrometer coupled to an Elemental Scientific Lasers NWR193 excimer laser ablation system with a TwoVol2 ablation chamber. Samples were analysed for ^7^Li, ^11^B, ^24^Mg, ^43^Ca, ^86^Sr, ^137^Ba, and ^238^U within the same analytical session to enable calculation of E/Ca. Samples and standards were ablated in line-mode. Standard analyses consisted of ca. 250 integration cycles of 0.47 s (1.2 mm lines), whilst sample analyses—which ablated the same area as analysed by LA-MC-ICP-MS (20 adjacent ca. 2 mm long ablation transects, again with a laser beam size of 25 μm^2^), consisted of ~ 433 integration cycles of 0.47 s. An estimated ≤ 10 µm of material is removed per laser pass based on the change in laser z-focal point, therefore the E/Ca images will be offset by a small degree to that of the δ^11^B image. Operating conditions are detailed in Supplementary Table S5. On-peak blank corrections were applied based on the mean intensities of preceding and succeeding blank measurements. Instrumental drift and mass bias were corrected by standard-sample bracketing with a pellet of JCp-1 and the values from the interlaboratory comparison study of Hathorne et al*.*^64^. Standard and sample data were screened and cycles falling outside of 2 SD (standards) or 4 SD (samples) of the mean were removed.

In-house reference material PS69/318-1b was ablated throughout the analytical session as a guide to internal precision, external reproducibility, and accuracy (Supplementary Table S6). Internal precision, expressed as 2 SE of the mean of the total number of integration cycles, was ≤ 5% for all ratios except Ba/Ca and U/Ca, which were ≤ 10%. External reproducibility, expressed as 2 SD of the mean of 5 analyses, was ≤ 5% for B/Ca, Mg/Ca, and Sr/Ca, and ≤ 16% for Li/Ca, Ba/Ca and U/Ca. Mean accuracy of all ratios is to within 10% relative to measurements by solution ICP-MS at the University of Southampton.

In-house reference material PS69/318-1b is a replacement for the in-house reference material PS69/318-1. Both are fragments of cold-water calcitic scleraxonian octocoral from the Pacific Sector of the Southern Ocean (depth of 1480–1788 m). Whilst the geochemical composition of PS69/318-1 has previously been published^66,67^, fragment PS69/318-1b was analysed by solution ICP-MS (element ratios) and MC-ICP-MS (boron isotope ratio) following standard methodologies (summarised in Standish et al.^67^). Mean values of three repeat analyses are reported in Supplementary Table S7.

### Calculation of pH and carbonate chemistry of the calcifying fluid

δ^11^B of marine carbonates (δ^11^B_CaCO3_) is related to pH of seawater (pH_sw_) by the following Eq. ^71^:

1 $${pH}_{sw}{= pK}_{B}-log\left(-\frac{{\delta }^{11}{B}_{sw}-{\delta }^{11}{B}_{CaCO3}}{{\delta }^{11}{B}_{sw}-{\alpha }_{B}\times {\delta }^{11}{B}_{CaCO3}-1000\times \left({\alpha }_{B}-1\right)}\right)$$

where δ^11^B_sw_ is the isotopic composition of seawater (39.61‰^72^), pK_B_ is calculated based on the measured temperature and salinity (T = 3.65 °C, S = 34.9; Dickson^73^), and the isotopic fractionation factor (α_B_) = 1.0272 (Klochko et al.^74^). For corals, this pH is thought instead to represent calcifying fluid pH, rather than that of seawater^44,75^.

Carbonate ion of the calcifying fluid [CO_3_^2−^] (in µmol/kg) was calculated for each sample using the following equation from DeCarlo et al.^76^ and based on work by Holcomb et al.^60^:

2 $${\left[{CO}_{3}^{2-}\right]}_{cf}=0.00297 \times \left(\frac{{\left[B{\left(OH\right)}_{4}^{-}\right]}_{cf}}{{B/Ca}_{m}}\times {10}^{6}\right)$$

where B/Ca is in µmol/mol and [B(OH)_4_^−^]_cf_ (in µmol/kg) is calculated following Dickson^73^:

3 $${\left[B{\left(OH\right)}_{4}^{-}\right]}_{cf}=\frac{{\left[B\right]}_{sw}}{1+{\left[H\right]}^{+}/{K}_{B}}$$

There are several other ways to calculate [CO_3_^2−^]_cf_ from paired δ^11^B and B/Ca measurements (see a detailed exploration in DeCarlo et al.^76^). We chose this method because it could easily be implemented within the Raster package in R^77^ to readily allow a 2D map to be constructed (Fig. 2). The alternative approaches do change the absolute values of [CO_3_^2−^]_cf_ estimated (e.g. Fietzke and Wall^39^), but do not change the relative patterns we observe between the structural components.

Dissolved inorganic carbonate [DIC]_cf_ of the calcifying fluid (in µmol/kg) was calculated from the pH_cf_, [CO_3_^2−^]_cf_, and mean measured water salinities (g/kg) and temperatures (°C) (Supplementary Table S1) in R using the seacarb package v.3.3.0^78^. Saturation state (Ω_cf_) was calculated following Zeebe and Wolf-Gladrow^71^:

4 $${\Omega }_{cf}= \frac{\left(\left[{Ca}^{2+}\right]\times \left[{CO}_{3}^{2-}\right]\right)}{{K}_{sp}}$$

where K_sp_ is the solubility product for aragonite, and [Ca^2+^] and [CO_3_^2−^] are the seawater calcium and carbonate ion concentrations, respectively. K_sp_ is calculated in R using the seacarb package v.3.3.0^78^, [CO_3_^2−^] is calculated from the B/Ca as per Eq. (2), and [Ca^2+^] is 10.28 mmol/kg^79^.

### Rayleigh-type fractionation model

Theoretical closed system Rayleigh-type fractionation models were calculated using the following Eqs. ^16^:

5 $${\left(\frac{X}{Ca}\right)}_{coral}= {D}_{x}{\left(\frac{X}{Ca}\right)}_{sol0}{F}^{{D}_{X}-1}$$

where the extent of precipitation is defined as:

6 $$F= {\left(\frac{\left[Ca\right]}{{\left[Ca\right]}_{0}}\right)}_{sol}$$

For fibrous aragonite, D_Li_ = 2.2 × 10^–3^, D_B_ = 1.2 × 10^–2^, D_Mg_ = 2.7 × 10^–4^, D_Sr_ = 1.22, D_Ba_ = 4.0, and D_U_ = 2.5; for COC, D_Li_ = 3.9 × 10^–3^, D_B_ = 8.8 × 10^–3^, D_Mg_ = 4.2 × 10^–4^, D_Sr_ = 1.28, D_Ba_ = 5.0, and D_U_ = 0.8. Calcifying fluid assumptions are as follows: [Li]sol0 = 26.0 μM, [B]sol0 = 433.3 μM, [Mg] sol0 = 52.9 mM, [Ca] sol0 = 10.3 mM, [Sr] sol0 = 91 μM, [Ba]sol0 = 0.05 μM, and [U]sol0 = 12.4 nM. Supplementary Fig.S4 shows the results for all elements.

### Isotopic and elemental mapping

The construction of isotopic and elemental maps was performed in R using an adapted script from Chalk et al.^36^ and using the unscreened data. Each laser line for Li/Ca, B/Ca, Mg/Ca, Sr/Ca, Ba/Ca, U/Ca and δ^11^B was subjected to a 5 SD rejection for the element to Ca ratio and 4 SD for the δ^11^B to remove any outliers, before a moving average was used to smooth the data. The width of the moving average window was 3 points for both elements and δ^11^B. The sets of 20 smoothed laser lines were mapped onto an equal spaced grid using their X and Y spatial coordinates from Elemental Scientific Lasers NWR193 excimer laser ablation system using the Raster package^77^. The X-Y resolution of the images produced was approximately 4.7 × 23.75 microns per pixel for the element data and 10.6 by 23.75 microns for the δ^11^B.

### Raman spectroscopy and coherent anti-stokes Raman microscopy (CARS)

Raman spectroscopy is a well-documented chemical spectroscopy technique with many databases of spectra for comparison and a well-defined theory to explore molecular scattering interactions^80^. Briefly, it is a chemical ‘fingerprinting’ technique that takes advantage of Raman scattering to probe material properties. Raman scattering results from interactions of photons with molecular vibrations and phonons and thus provides characteristic chemical and structural information about a material.

Raman micro-spectroscopy experiments within this work were conducted using a Renishaw InVia Raman microscope (Renishaw, UK), with a Leica DM 2500-M bright field microscope and an automated 100 nm-encoded XYZ stage. Samples were excited using a 532 nm laser directed through a Nikon 50X air objective (NA = 0.5), with collection after a Rayleigh edge filter (~ 100 cm^−1^ cut-off), and a diffraction grating (1800 lines/mm) to disperse the Raman-scattered light onto a Peltier-cooled charge coupled device (1024 pixels × 256 pixels). This results in a spectral resolution of 2.97 cm^−1^ measured by analysing the width of neon light source peaks. Raman shift calibration was carried out using an internal silicon wafer using the peak at 520 cm^−1^. Results of this are used to verify the presence of organic structures within the COC and allow us to target specific vibrational peaks with CARS. The results displayed in this paper were produced by scanning 400 points in a 20 × 20 grid with 1 μm spacing across the COC producing a mean spectrum.

CARS Microscopy allows for mapping chemical vibrational signatures in a fast-imaging platform. CARS imaging is well-used for the study of organic content by targeting the CH-stretching modes of vibration, a region of the Raman spectrum (2800–3100 cm^−1^) where organic material is normally labelled due to its high number of CH bonds^81,82^. CARS is a multiphoton interaction in which 2 photons (pump and Stokes photons) interact to generate a vibrational coherence in a material. A third photon then probes this coherence to generate an anti-Stokes shifted photon, which is detected. On tuning the difference between pump and Stokes wavelengths to match a vibrational resonance, the CARS signals are substantially enhanced and thus produce chemical-specific images when coupled to a laser scanning microscope^83^.

For this study, CARS microscopy in the single frequency imaging configuration was carried out on a multimodal imaging system: a Zeiss Laser Scanning Microscope (LSM) [LSM 980] coupled with an A.P.E Berlin Pico-Emerald Laser and using a 20 × 1 NA water dipping objective. The Pico-emerald laser allows for the generation of non-linear effects and has both a tuneable pump source (700–1300 nm) and a fixed stokes source at 1031.5 nm. By tuning the pump source, we can target specific vibrational modes revealed by Raman spectroscopy. We have shown one Raman peak at 2847 cm^−1^ that is assigned to CH_2_ stretching frequency and, within life sciences, is typically used as a marker for CARS imaging of lipids^84^. Within this study we use it as a marker for general organic content, additional C-H peaks can be seen in the micro-Raman data in Supplementary Fig. S4. Supplementary Fig. S5 shows a simplified schematic of our multimodal imaging system. Image acquisition is optimised for Nyquist sampling at 0.097 μm per pixel, with a theoretical full width half maximum lateral resolution at 0.306 μm.

CARS image acquisition required the imaging of sequential, smaller image subsets to expand the field of view to incorporate the entire ablation area. A total of 14 images (7*x, 2*y) were acquired and stitched using the Fiji plugin, Microscopy Image Stitching Tool (MIST^85^). Images were stitched and noise was removed using a despeckle filter followed by application of gaussian blur. A fast fourier transform (FFT) was then used to remove tiling artefacts.

### Correlative multimodal imaging

The acquired ICP-MS datasets were imported into Fiji^86^ for processing prior to alignment. Initially, element (Supplementary Fig. S6a, 7 × 2D images) and boron isotope heatmaps (Supplementary Fig. S6b, 1 × 2D image) required alignment. To eliminate surrounding blank space surrounding each image, a custom in-house Fiji auto crop tool was applied.

To provide distinct borders to aid dataset alignment, the K-Means clustering algorithm was employed to separate each δ^11^B and element image into four intensity clusters. A mean intensity image was then computed for the element stack (Supplementary Figure S6c), providing an outline of (1) the COC and (2) the boundary between the fibrous region and the secondary COCs. These two boundaries were also visible following clustering of the δ^11^B dataset (Supplementary Figure S6d), with the use of multiple shared features maximising the appearance of landmark features for use by subsequent alignment algorithms. This use of multiple shared features—versus aligning using a singular, manually segmented, feature such as the COC—reduces potential error which may arise due to variations in the boundary of the COC occurring between trace element maps, thus improving confidence in achieving an accurate multimodal alignment. Previously, the sequential usage of K-Means clustering to simplify feature input, followed by a mutual information algorithm has been described for the unsupervised alignment of multi-modal datasets within life sciences^87^.

The clustering outputs obtained from Fiji were imported into Dragonfly (2022.1, Comet Technologies Canada Inc, Montreal, Canada) for registration. To align the δ^11^B to the average element clustering outputs, Dragonfly's automated image registration tool was utilised, employing a rigid Maximisation of Mutual Information (MMI) algorithm^88^. The MMI algorithm varied the rotational and transformational positioning of the δ^11^B clustering image relative to the element clustering image which remained stationary. An initial, course step size of 2 µm (transformation) and 1° (rotation) was used followed by a subsequent fine step size of 0.01 µm and 0.01°. The raw δ^11^B image was then imported, and the transformation that was applied to the δ^11^B cluster during the prior MMI-based alignment was applied to it. The resulting δ^11^B dataset was resampled to match the geometry of the element stack, ensuring the elimination of non-shared pixels. The successful alignment of the δ^11^B and element datasets enabled the calculation of the calcifying fluid carbonate system. This calculation provided heatmaps for pH_cf_, [CO_3_^2−^]_cf_, Ω_cf_, and [DIC]_cf_.

Following this, the auto crop—K-Means clustering—stack averaging workflow was again utilised, before the resulting carbonate system clusters were aligned to the δ^11^B and element clusters, again, using a rigid MMI algorithm. Following the alignment of the carbonate system clusters to the elemental and δ^11^B datasets, clustering and z-averaging analysis was performed again on this full dataset to obtain an average clustered image that could be used to align other modalities (CARS, Optical) for further analysis.

To provide additional, optical context, a brightfield image of the pre-ablated area was imported into Fiji and clustering analysis applied. The clustered brightfield images were then imported into Dragonfly and aligned to the complete geochemical clusters using the MMI algorithm (Supplementary Figure S6e). This transformation was then applied to the raw optical image (Supplementary Figure S6f.). Once aligned, down-sampling was used to obtain pixel-by-pixel intensity values (0–255) written into CSV format, with each column of data representing each column of pixels. Greyscale values were then converted to scalar values for each modality based on the original measured values using a linear transformation. Reproducibility of the MMI algorithm, and therefore the automated correlative approach, was < 2 pixels in both the x and y dimensions based on comparing the centre of mass for 6 repeat alignments of the geochemistry images to the optical.

Three authors independently identified the primary COC, fibrous regions, and secondary COCs from the optical image (Supplementary Figure S6e) by manually filling areas using Dragonfly’s painter tool. An average image of the three segmentations was generated, with the proportion of pixels allocated by each individual author to each region quantified as a percentage of the total number of pixels. Mean percentages (± 1 SD) assigned to each structural group are as follows: 6.9 ± 0.6% for the primary COC, 43.1 ± 0.5% for the secondary COCs, and 49.9 ± 0.9% for the fibrous regions. This gives a mean inter-author variability of ± 0.68% between individual selections, which serves as an indicator of uncertainty for our segmentation approach. Each compartment was allocated a distinct pixel intensity; primary COC an intensity of 1, fibrous regions an intensity of 2, and secondary COC an intensity of 3, with the pixel intensities converted into CSV format. Following this, the interpretation of each CMI subcomponent (e.g. CARS, E/Ca, calcifying fluid carbonate system) was guided by this segmentation-based data sub-setting, allowing the compartmentalisation of CMI datasets for subsequent comparative analyses of the skeletal regions.

### Supplementary Information

Supplementary Information 1.

Supplementary Video 1.

Supplementary Information 2.

## Data Availability

All data generated or analysed during this study are included in this published article (and its Supplementary Information files).

## References

[1] Knowlton N (2001). The future of coral reefs. PNAS.

[2] de Groot R (2012). Global estimates of the value of ecosystems and their services in monetary units. Ecosyst. Serv..

[3] Roberts JM (2006). Reefs of the deep: The biology and geology of cold-water coral ecosystems. Science.

[4] Robinson LF (2014). The geochemistry of deep-sea coral skeletons: A review of vital effects and applications for palaeoceanography. Deep-Sea Res. Part.

[5] Drake JL (2020). How corals made rocks through the ages. Global Change Biol..

[6] Gilbert P (2022). Biomineralization: Integrating mechanism and evolutionary history. Sci. Adv..

[7] Mass T (2017). Amorphous calcium carbonate particles form coral skeletons. PNAS.

[8] Cohen AL, McConnaughey TA (2003). Geochemical perspectives on coral mineralization. Rev. Mineral. Geochem..

[9] Sun C-Y (2020). From particle attachment to space-filling coral skeletons. PNAS.

[10] DeCarlo TM (2019). Investigating marine bio-calcification mechanisms in a changing ocean with in vivo and high-resolution ex vivo Raman spectroscopy. Glob. Change Biol..

[11] Nothdurft LD, Webb GE (2007). Microstructure of common reef-building coral genera *Acropora*, *Pocillopora*, *Goniastrea* and *Porites*: Constraints on spatial resolution in geochemical sampling. Facies.

[12] Cuif J-P (2003). XANES mapping of organic sulfate in three scleractinian coral skeletons. Geochim. Cosmochim..

[13] Meibom A (2004). Distribution of magnesium in coral skeleton. Geophys. Res. Lett..

[14] Gladfelter EH (2007). Skeletal development in *Acropora palmata* (Lamarck 1816): a scanning electron microscope (SEM) comparison demonstrating similar mechanisms of skeletal extension in axial versus encrusting growth. Coral Reefs.

[15] Allison N, Finch AA (2007). High temporal resolution Mg/Ca and Ba/Ca records in modern *Porites lobata* corals. Geochem. Geophys. Geosyst..

[16] Gagnon AC (2007). Sr/Ca and Mg/Ca vital effects correlated with skeletal architecture in a scleractinian deep-sea coral and the role of Rayleigh fractionation. Earth Planet. Sci. Lett..

[17] Case DH (2010). Environmental and biological controls on Mg and Li in deep-sea scleractinian corals. Earth Planet. Sci. Lett..

[18] Rollion-Bard C, Blamart D (2015). Possible controls on Li, Na, and Mg incorporation into aragonite coral skeletons. Chem. Geol..

[19] Stewart JA (2016). An improved boron isotope pH proxy calibration for the deep-sea coral *Desmophyllum dianthus* through sub-sampling of fibrous aragonite. Chem. Geol..

[20] Chen S (2023). Coherent tracer correlations in deep-sea corals and implications for biomineralization mechanisms underlying vital effects. Geochim. Cosmochim. Acta.

[21] Cohen AL (2001). Kinetic control of skeletal Sr/Ca in a symbiotic coral: Implications for the paleotemperature proxy. Paleoceanogr. Paleoclimatol..

[22] Allison N, Finch AA (2004). High-resolution Sr/Ca records in modern Porites lobata corals: Effects of skeletal extension rate and architecture. Geochem. Geophys. Geosyst..

[23] Allison N (2005). Strontium in coral aragonite: 3. Sr co-ordination in relation to skeletal architecture. Geochim. Cosmochim. Acta.

[24] Shirai K (2005). Deep-sea coral geochemistry: Implication for the vital effect. Chem. Geol..

[25] Sinclair DJ (2006). A biological origin for climate signals in corals—Trace element “vital effects” are ubiquitous in Scleractinian coral skeletons. Geophys. Res. Lett..

[26] Blamart D (2007). Correlation of boron isotopic composition with ultrastructure in the deep-sea coral *Lophelia pertusa*: Implications for biomineralization and paleo-pH. Geochem. Geophys. Geosyst..

[27] Jurikova H (2019). Boron isotope composition of the cold-water coral Lophelia pertusa along the Norwegian margin: Zooming into a potential pH-proxy by combing bulk and high-resolution approaches. Chem. Geol..

[28] Adkins JF (2003). Stable isotopes in deep-sea corals and a new mechanism for “vital effects”. Geochim. Cosmochim. Acta.

[29] Robinson LF (2006). Primary U distribution in scleractinian corals and its implications for U series dating. Geochem. Geophys. Geosyst..

[30] Lazier AV (2007). The skeletal structure of *Desmophyllum cristagalli*: The use of deep-water corals in sclerochronology. Lethaia.

[31] Smith JE (2002). Patterns of isotopic disequilibria in azooxanthellate coral skeletons. Hydrobiologia.

[32] Anagnostou E (2012). Evaluation of boron isotope ratio as a pH proxy in the deep sea coral *Desmophyllum dianthus*: Evidence of physiological pH adjustment. Earth Planet. Sci. Lett..

[33] Sinclair DJ (2005). Correlated trace element “vital effects” in tropical corals: A new geochemical tool for probing biomineralization. Geochim. Cosmochim. Acta.

[34] Karreman MA (2016). Fast and precise targeting of single tumor cells in vivo by multimodal correlative microscopy. J. Cell Sci..

[35] Zopf LM (2021). Cross-modality imaging of murine tumor vasculature—A feasibility study. Mol. Imag. Biol..

[36] Chalk TB (2021). Mapping coral calcification strategies from in situ boron isotope and trace element measurements of the tropical coral *Siderastrea siderea*. Sci. Rep..

[37] Chew D (2021). LA-ICP-MS imaging in the geosciences and its applications to geochronology. Chem. Geol..

[38] Sliwinski JT, Stoll HM (2021). Combined fluorescence imaging and LA-ICP-MS trace element mapping of stalagmites: Microfabric identification and interpretation. Chem. Geol..

[39] Fietzke J, Wall M (2022). Distinct fine-scale variations in calcification control revealed by high-resolution 2D boron laser images in the cold-water coral *Lophelia pertusa*. Sci. Adv..

[40] McCulloch M (2017). Coral calcification in a changing World and the interactive dynamics of pH and DIC upregulation. Nat. Commun..

[41] Stewart JA (2020). Refining trace metal temperature proxies in cold-water scleractinian and stylasterid corals. Earth Planet. Sci. Lett..

[42] Kershaw J (2023). Ba/Ca of stylasterid coral skeletons records dissolved seawater barium concentrations. Chem. Geol..

[43] DeCarlo TM (2018). The origin and role of organic matrix in coral calcification: Insights from comparing coral skeleton and abiogenic aragonite. Front. Mar. Sci..

[44] Holcomb M (2014). Coral calcifying fluid pH dictates response to ocean acidification. Sci. Rep..

[45] D’Olivio JP, McCulloch MT (2017). Response of coral calcification and calcifying fluid composition to thermally induced bleaching stress. Sci. Rep..

[46] Allison N (2023). A comparison of SNARF-1 and skeletal δ11B estimates of calcification media pH in tropical coral. Geochim. Cosmochim. Acta.

[47] Allison N (2017). Reconstructing coral calcification fluid dissolved inorganic carbon chemistry from skeletal boron: An exploration of potential controls on coral aragonite B/Ca. Heliyon.

[48] DeCarlo TM (2015). Experimental determination of factors controlling U/Ca of aragonite precipitated from seawater: Implications for interpreting coral skeleton. Geochim. Cosmochim. Acta.

[49] Guo W (2019). Seawater temperature and buffering capacity modulate coral calcifying pH. Sci. Rep..

[50] Bertucci A (2013). Carbonic anhydrases in anthozoan corals—A review. Bioorg. Med. Chem..

[51] Addadi L (2003). Taking advantage of disorder: Amorphous calcium carbonate and its roles in biomineralization. Adv. Mater..

[52] Weiner S (2003). Biologically formed amorphous calcium carbonate. Connect. Tissue Res..

[53] Politi Y (2010). Role of magnesium ion in the stabilization of biogenic amorphous calcium carbonate: A structure−function investigation. Chem. Mater..

[54] Reggi M (2014). Biomineralization in Mediterranean corals: The role of the intraskeletal organic matrix. Cryst. Growth Des..

[55] DeVol RT (2015). Nanoscale transforming mineral phases in fresh nacre. J. Am. Chem. Soc..

[56] Von Euw S (2017). Biological control of aragonite formation in stony corals. Science.

[57] Gagnon AC (2021). Controls on boron isotopes in a cold-water coral and the cost of resilience to ocean acidification. Earth Planet. Sci. Lett..

[58] Mavromatis V (2021). Boron isotope fractionation during the formation of amorphous calcium carbonates and their transformation to Mg-calcite and aragonite. Geochim. Cosmochim. Acta.

[59] Henehan MJ (2022). No ion is an island: Multiple ions influence boron incorporation into CaCO_3_. Geochim. Cosmochim. Acta.

[60] Holcomb M (2016). Factors affecting B/Ca ratios in synthetic aragonite. Chem. Geol..

[61] Evans D (2022). Trace and major element incorporation into amorphous calcium carbonate (ACC) precipitated from seawater. Geochim. Cosmochim. Acta.

[62] Gaetani GA, Cohen AL (2006). Element partitioning during precipitation of aragonite from seawater: A framework for understanding paleoproxies. Geochim. Cosmochim. Acta.

[63] Tambutté E (2015). Morphological plasticity of the coral skeleton under CO_2_-driven seawater acidification. Nat. Commun..

[64] Hathorne EC (2013). Interlaboratory study for coral Sr/Ca and other element/Ca ratio measurements. Geochem. Geophys. Geosyst..

[65] Stewart JA (2021). NIST RM 8301 boron isotopes in marine carbonate (simulated coral and foraminifera solutions): Inter-laboratory δ11B and trace element ratio value assignment. Geostand. Geoanal. Res..

[66] Foster GL (2013). Interlaboratory comparison of boron isotope analyses of boric acid, seawater and marine CaCO_3_ by MC-ICPMS and NTIMS. Chem. Geol..

[67] Standish CD (2019). The effect of matrix interferences on in situ boron isotope analysis by laser ablation multi-collector inductively coupled plasma mass spectrometry. Rapid Commun. Mass Spectrom..

[68] Evans D (2021). Accurate correction for the matrix interference on laser ablation MC-ICPMS boron isotope measurements in CaCO_3_ and silicate matrices. J. Anal. At. Spectrom..

[69] Inoue M (2004). Concentrations of trace elements in carbonate reference materials coral JCp-1 and giant clam JCt-1 by inductively coupled plasma-mass spectrometry. Geostand. Geoanal. Res..

[70] Gutjahr M (2021). Sub-permil interlaboratory consistency for solution-based boron isotope analyses on marine carbonates. Geostand. Geoanal. Res..

[71] Zeebe RE, Wolf-Gladrow DA (2001). CO2 in Seawater: Equilibrium, Kinetics, Isotopes.

[72] Foster GL (2010). Boron and magnesium isotopic composition of seawater. Geochem. Geophys. Geosyst..

[73] Dickson AG (1990). Thermodynamics of the dissociation of boric acid in synthetic seawater from 273.15 to 318.15 K. Deep Sea Res. Part..

[74] Klochko K (2006). Experimental measurement of boron isotope fractionation in seawater. Earth Planet. Sci. Lett..

[75] Krief S (2010). Physiological and isotopic responses of scleractinian corals to ocean acidification. Geochim. Cosmochim. Acta.

[76] DeCarlo TM (2018). Reviews and syntheses: Revisiting the boron systematics of aragonite and their application to coral calcification. Biogeosciences.

[77] Hijmans, R., Van Etten, J. Geographic analysis and modelling with raster data. R Package Version 2, 1–25; http://raster.r-forge.r-project.org/ (2012).

[78] Gattuso, J. -P. *et al*. Package ‘seacarb’: Seawater carbonate chemistry. R package version 3.3.0. https://CRAN.R-project.org/package=seacarb (2021).

[79] Millero FJ (2008). The composition of standard Seawater and the definition of the reference-composition salinity scale. Deep Sea Res. Part.

[80] Smith E, Dent G (2019). Modern Raman spectroscopy: A practical approach.

[81] Patel II (2013). Coherent anti-Stokes Raman scattering for label-free biomedical imaging. J. Opt..

[82] Smus JP (2017). Coherent anti-Stokes Raman scattering (CARS) spectroscopy in *Caenorhabditis elegans* and *Globodera pallida*: Evidence for an ivermectin-activated decrease in lipid stores. Pest Manag. Sci..

[83] Cheng JX, Xie XS (2016). Coherent Raman scattering. Microscopy.

[84] Yu Y (1841). Shedding new light on lipid functions with CARS and SRS microscopy. Biochim. Biophys. Acta.

[85] Chalfoun J (2017). MIST: Accurate and scalable microscopy image stitching tool with stage modeling and error minimization. Sci. Rep..

[86] Schindelin J (2012). Fiji: An open-source platform for biological-image analysis. Nat. Methods.

[87] Öfverstedt J (2022). Fast computation of mutual information in the frequency domain with applications to global multimodal image alignment. Pattern Recognit. Lett..

[88] Maes F (1997). Multimodality image registration by maximization of mutual information. IEEE Trans. Med. Imaging.

[89] Olsen A (2020). An updated version of the global interior ocean biogeochemical data product, GLODAPv2.2020. Earth Syst. Sci. Data Discuss..

[90] Cohen J (1988). Statistical Power Analysis for the Behavioral Sciences.

