# Supplementary material for: Correlative geochemical imaging of Desmophyllum dianthus reveals biomineralisation strategy as a key coral vital effect
Source: Sci Rep. 2024 May 15;14:11121. doi: 10.1038/s41598-024-61772-2 (PMC11096413; doi:10.1038/s41598-024-61772-2)
Supplement: Supplementary file 3 — Supplementary Information 2. [file 41598_2024_61772_MOESM3_ESM.docx]

**Correlative geochemical imaging of *Desmophyllum dianthus* reveals biomineralisation strategy as a key coral vital effect**

Christopher D. Standish^1*^, Jacob Trend^1^, Jacob Kleboe^2^, Thomas B. Chalk^1,3^, Sumeet Mahajan^2^, J. Andy Milton^1^, Tessa M. Page^1^, Laura F. Robinson^4^, Joseph A. Stewart^4^, Gavin L. Foster^1^

^1^School of Ocean & Earth Sciences, University of Southampton, National Oceanography

Centre, European Way, Southampton, SO14 3ZH, UK.

^2^Department of Chemistry and Institute for Life Sciences, University of Southampton, Highfield Campus, University Road, Southampton, SO17 1BJ, UK.

^3^Aix Marseille Université, CNRS, IRD, INRAE, Coll France, CEREGE, Aix-en-Provence, France.

^4^School of Earth Sciences, University of Bristol, Queens Road, Bristol BS8 1RJ, UK.

^*^Corresponding author (email: c.d.standish@soton.ac.uk)

# Supplementary Information

## SM Figures


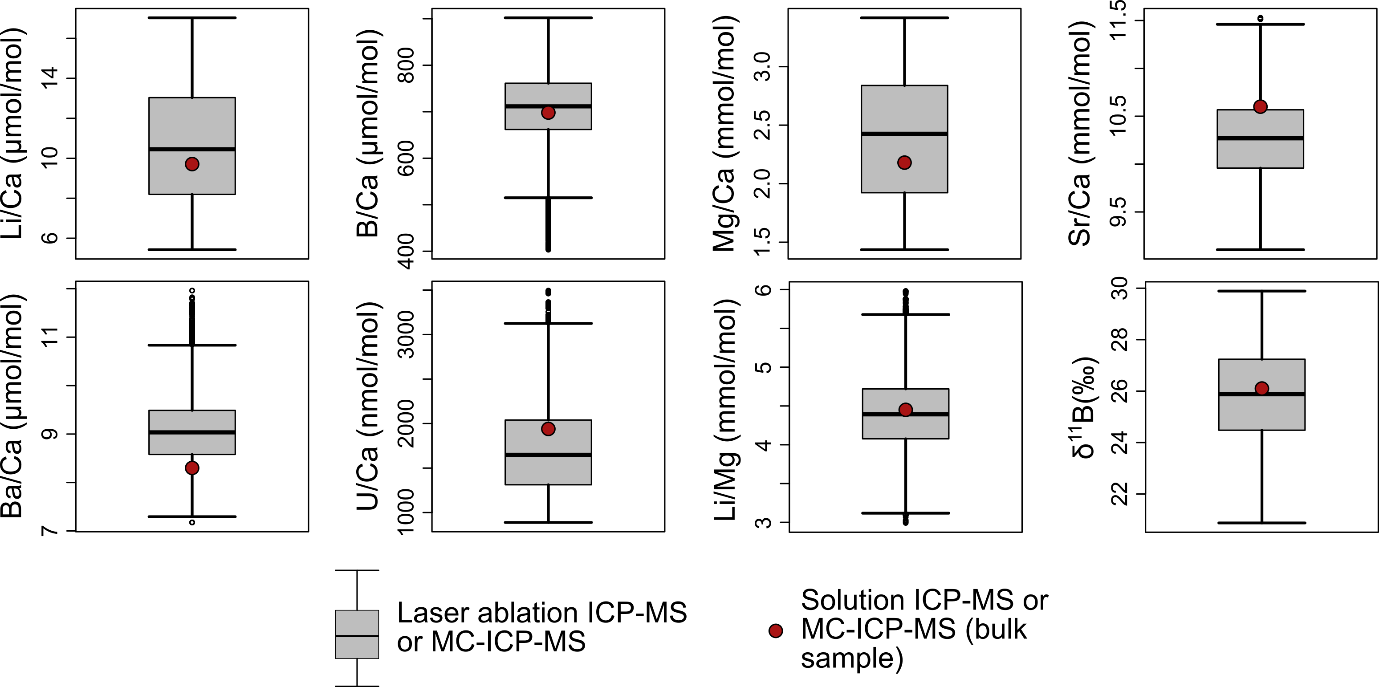


Figure S1. Boxplots of DY081-914DD LA-(MC)-ICP-MS analyses compared to analyses by solution (MC)-ICP-MS (red data point) from Stewart *et al*.^41^, Kershaw *et al*.^42^, and this study.


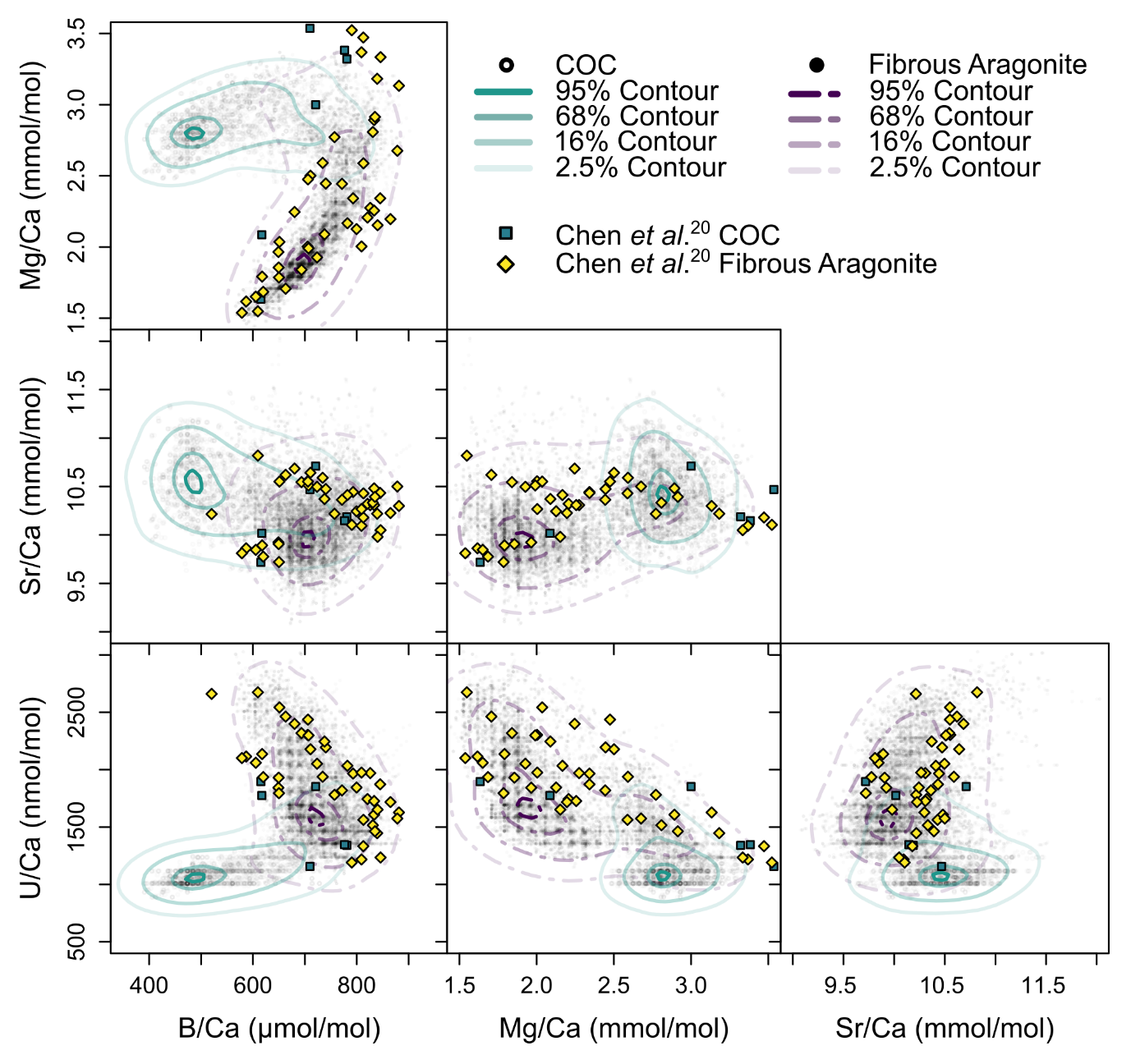
 Figure S2. Cross plots of selected E/Ca for DY081-914DD (black data points) compared to data from Chen *et al*.^20^. Data from this study are contoured at confidence levels of 95%, 68%, 16% and 2.5% respectively (COC with solid turquoise lines, fibrous aragonite with dashed purple lines). Data from Chen *et al*.^20^ are plotted as green squares (COC) and yellow diamonds (fibrous aragonite). Note how for most elements there is considerable overlap in the Chen *et al*.^20^ data between COC and fibre attributed in that study to inadvertent mixing of these components during sampling.


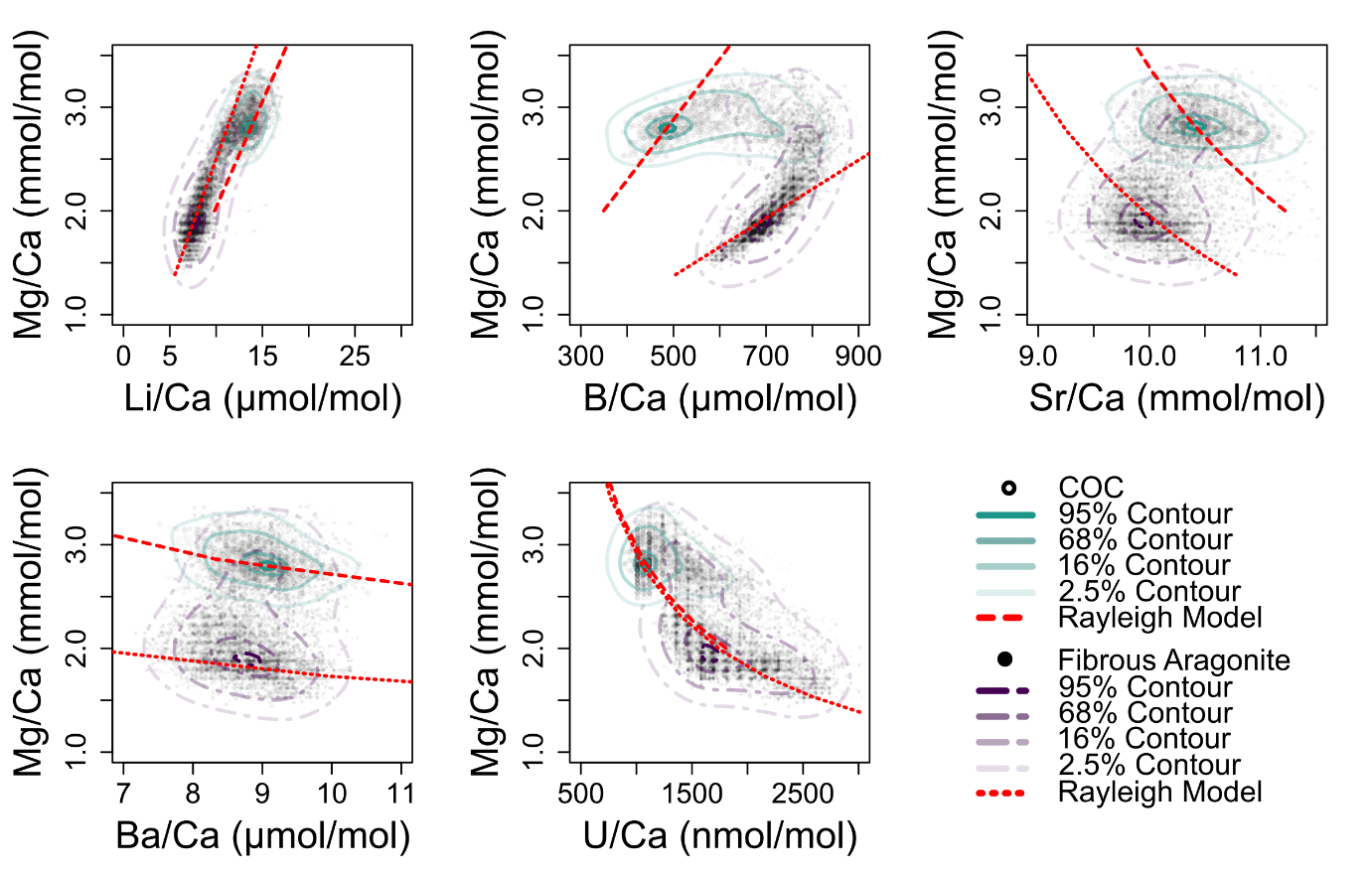


Figure S3. Rayleigh fractionation models for Li, B, Mg, Sr, Ba and U incorporation into the aragonitic skeleton of DY081-914DD (black data points). Data for the COC and fibrous aragonite are contoured at confidence levels of 95%, 68%, 16% and 2.5% respectively.


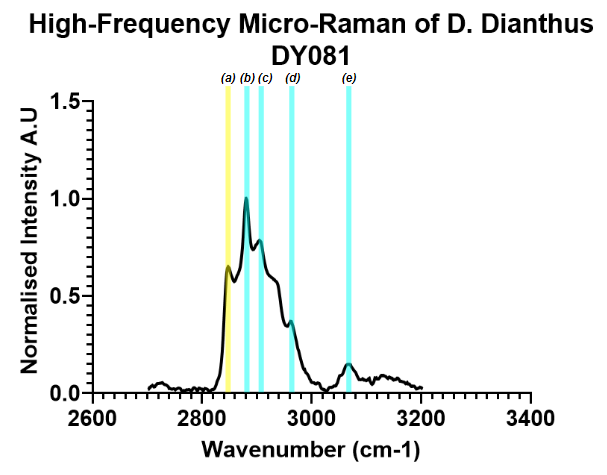


Figure S4. High-frequency micro-Raman of *D. Dianthus* DY081-914DD. Micro-Raman data was taken across 400 locations in mapping mode across the COC before smoothing, normalising to maximum intensity, and averaging the spectra (400 locations, 3 spectra average per point). Multiple peaks can be seen in the spectra, suggesting several CH-stretching modes of vibrations. In this study, CARS microscopy was targeted on the 2847 cm^-1^ peak (a), but several other peaks are present in the Raman spectra: (b) 2880 cm^-1^ (c) 2907 cm^-1^ (d) 2962 cm^-1^ (e) 3067 cm^-1^. Peak positions were found by curve fitting the average spectrum within the Renishaw Wire 5.5 software. CARS images at 2847cm^-1^ were chosen as a valid approximation of organics for comparison to other modes in this CMI methodology.


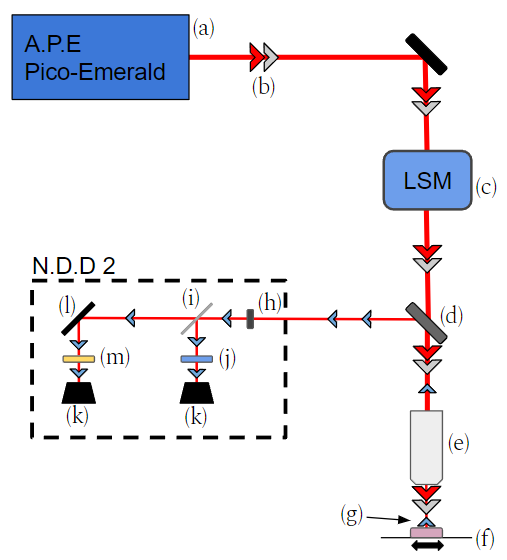


Figure S5. (a) Pico-Emerald S Laser producing tuneable pump light source (700nm–1300nm) and fixed 1031nm stokes light source. (b) Spatially overlapped pump-Stokes collinear beams are coupled into the laser-scanning microscope. (c) Scan mirror system within microscope head. (d) Removable 760nm++ dichroic to direct the backscattered signal towards the detector. (e) 20x 1.0 NA water dipping objective. (g) Backscattered light is collected from the same objective under epi-illumination configuration. (f) Axio-examiner z-stage for placing the sample (h) 760++ SP filter to filter any remnant laser scatter (i) <555nm LP to direct CARS and 2-Photon signals to PMTs. (j) 500–550nm BP to filter 2-Photon signals. (k) Multi-alkaline PMT’s. (l) Mirror to direct CARS signals to PMT. (m) 650+-20nm BP (FBH650-40, Thor labs) to filter CARS signals.


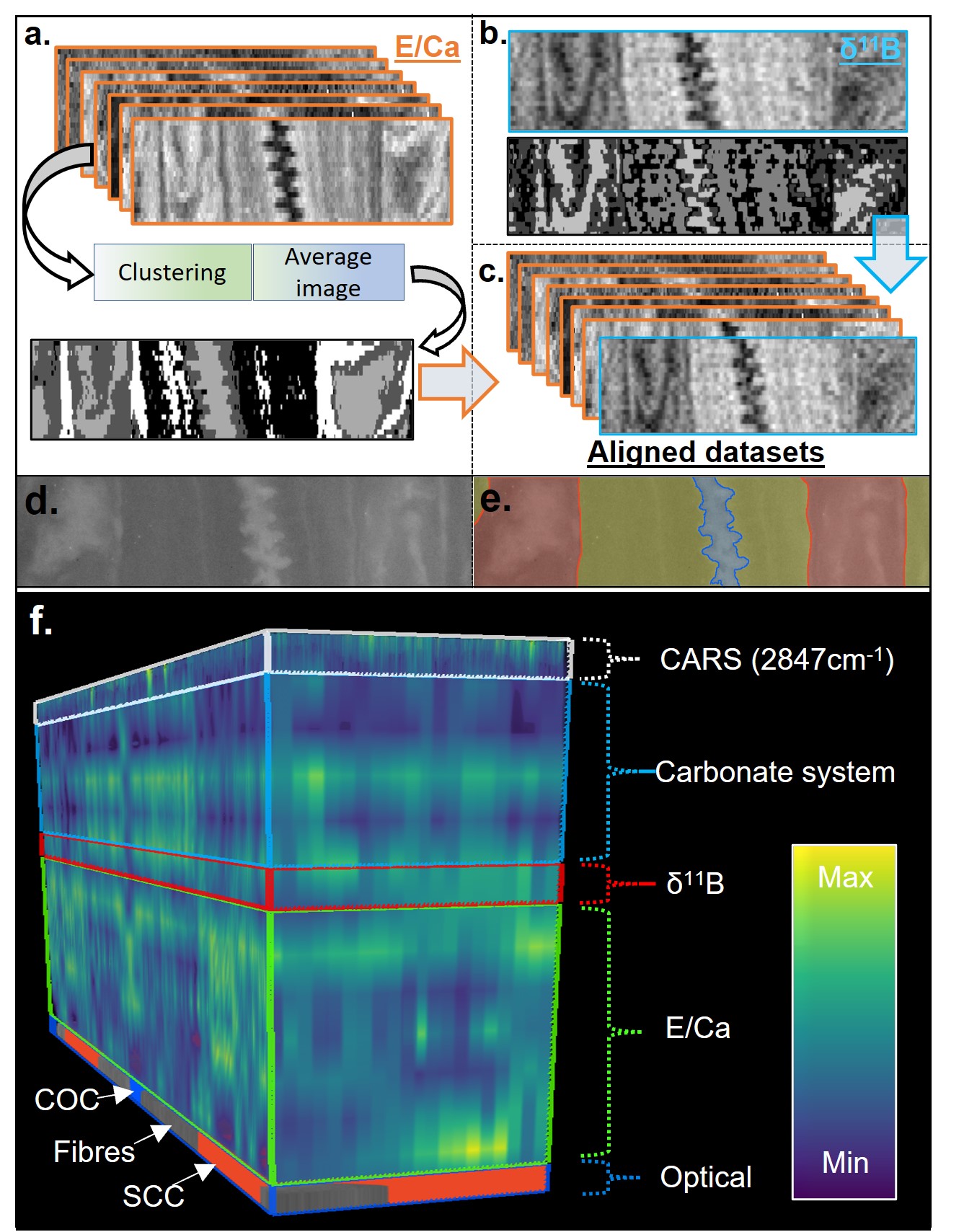


Figure S6. Correlative multimodal imaging workflow. Greyscale stacks of element/Ca (a) and δ^11^B (b) images were clustered using the K-Means Clustering algorithm. The two clustering images were aligned using MMI, and the MMI-derived transforms from the alignment of clustered images were applied to the greyscale E/Ca and δ^11^B images (c). This workflow was utilised to align optical and CARS datasets. A brightfield image was taken of the ablation area (d) and segmented by 3 independent investigators to separate the COC (blue), fibres (yellow) and SCC (red) (e). This workflow permitted the correlative, pixel-by-pixel quantification of geochemical and CARS images, with sub-regions identified through segmentation of the optical image (f).

## SM Tables

LA-(MC)-ICP-MS and CARS data: see supplementary data file.

Table S1. Hydrographic data relating to sample DY081-914DD. Temperature, salinity, and pressure are from Stewart *et al*.^41^ based on shipboard CTD and seawater bottle measurements on Cruise DY081. Temperature, pH, and δ^11^B_borate_ uncertainty are calculated using 2 standard deviations of these shipboard measurements and two other nearby seawater bottle measurements from the GLODAP database^89^

| Temperature (°C) | 3.65 |
| --- | --- |
| Temperature 2SD (°C) | 0.32 |
| Salinity | 34.90 |
| Pressure (dBar) | 1067 |
| Alkalinity (µmol/kg) | 2289.2 |
| DIC (µmol/kg) | 2168.4 |
| pH_Total_ | 7.94 |
| pH_Total_ 2SD | 0.04 |
| pK_B_ | 8.80 |
| δ^11^B_borate_ (‰) | 15.32 |
| δ^11^B_borate_ 2SD (‰) | 0.36 |

Table S2. Bulk geochemical composition of DY081-914DD determined by solution ICP-MS and solution MC-ICP-MS.

|  |  | Replicate #1 | Replicate #2 | Mean | 2SD | Reference |
| --- | --- | --- | --- | --- | --- | --- |
| Li/Ca | (µmol/mol) | 10.02 | 9.41 | 9.71 | 0.85 | Stewart *et al*.^41^ |
| **B/Ca** | **(µmol/mol)** | **722.10** | **674.24** | **698.17** | **67.68** | **This Study** |
| Mg/Ca | (mmol/mol) | 2.23 | 2.14 | 2.18 | 0.13 | Stewart *et al*.^41^ |
| Mn/Ca | (µmol/mol) | 0.14 | 0.15 | 0.15 | 0.01 | Kershaw *et al*.^42^ |
| Sr/Ca | (mmol/mol) | 10.58 | 10.63 | 10.60 | 0.06 | Stewart *et al*.^41^ |
| Ba/Ca | (µmol/mol) | 8.51 | 8.08 | 8.30 | 0.60 | Kershaw *et al*.^42^ |
| **U/Ca** | **(nmol/mol)** | **1977.33** | **1871.63** | **1924.48** | **149.48** | **This Study** |
| Fe/Ca | (µmol/mol) | <LOD | <LOD | - | - | Kershaw *et al*.^42^ |
| Li/Mg | (mmol/mol) | 4.49 | 4.41 | 4.45 | 0.12 | Stewart *et al*.^41^ |
| **δ^11^B** | **(‰)** | **26.23** | **25.97** | **26.10** | **0.36** | **This Study** |

Table S3. Geochemical composition of DY081-914DD determined by LA-ICP-MS and LA-MC-ICP-MS.

|  |  | **CMI All Data** | | **CMI COC** | | **CMI Fibre** | | **CMI Secondary COC** | | |
| --- | --- | --- | --- | --- | --- | --- | --- | --- | --- | --- |
|  |  | **Mean** | **SD** | **Mean** | **SD** | **Mean** | **SD** | | **Mean** | **SD** |
| **Li/Ca** | (µmol/mol) | 10.68 | 2.70 | 13.29 | 1.27 | 8.94 | 2.05 | | 11.77 | 2.18 |
| **B/Ca** | (µmol/mol) | 706.28 | 79.74 | 563.32 | 100.51 | 717.26 | 58.37 | | 723.66 | 76.09 |
| **Mg/Ca** | (mmol/mol) | 2.40 | 0.49 | 2.85 | 0.16 | 2.15 | 0.42 | | 2.57 | 0.43 |
| **Sr/Ca** | (mmol/mol) | 10.27 | 0.40 | 10.46 | 0.33 | 10.10 | 0.33 | | 10.73 | 0.37 |
| **Ba/Ca** | (µmol/mol) | 9.05 | 0.68 | 8.96 | 0.48 | 8.81 | 0.56 | | 9.47 | 0.62 |
| **U/Ca** | (nmol/mol) | 1715.63 | 497.63 | 1162.96 | 199.82 | 1787.43 | 391.63 | | 1793.86 | 622.47 |
| **Li/Mg** | (mmol/mol) | 4.40 | 0.47 | 4.69 | 0.45 | 4.10 | 0.43 | | 4.58 | 0.42 |
| **CARS** | (a.u.) | 7343.64 | 3847.44 | 9196.60 | 5466.89 | 6513.47 | 2861.71 | | 7985.59 | 4238.60 |
| **δ^11^B** | (‰) | 25.80 | 1.71 | 24.27 | 1.56 | 27.02 | 1.18 | | 24.76 | 1.19 |
| **pH** | - | 8.78 | 0.12 | 8.67 | 0.11 | 8.86 | 0.08 | | 8.70 | 0.08 |
| **[DIC]** | (µmol/kg) | 3802.8 | 666.0 | 5182.9 | 1002.6 | 3531.0 | 390.2 | | 3888.9 | 526.1 |
| **[CO_3_^2-^]** | (µmol/kg) | 883.1 | 132.2 | 965.5 | 159.5 | 954.2 | 96.6 | | 786.8 | 97.1 |
| **Ω_arag_** | - | 11.42 | 1.71 | 12.48 | 2.06 | 12.34 | 1.25 | | 10.17 | 1.26 |

## Table S4. Results of Cohen’s d Effect Size comparing DY081-914DD COC with fibrous aragonite. Following the benchmarks suggested by Cohen^90^, d-values of <0.2 are interpreted as reflecting negligible effect sizes (i.e. difference between two population means), values of 0.2–0.5 are interpreted as reflecting small effect sizes, values of 0.5–0.8 are interpreted as medium effect sizes, and values of >0.8 are interpreted as large effect sizes. Positive values reflect that the mean of the COC is higher than the fibrous aragonite, negative values reflect that the mean of the COC is lower than the fibrous aragonite.

| **Parameter** | **d-value** |
| --- | --- |
| **Li/Ca** | 2.21 |
| **B/Ca** | -2.36 |
| **Mg/Ca** | 1.76 |
| **Sr/Ca** | 1.07 |
| **Ba/Ca** | 0.28 |
| **U/Ca** | -1.67 |
| **Li/Mg** | 1.35 |
| **δ^11^B** | -2.23 |
| **Organic Content** | 0.81 |
| **pH** | -2.26 |
| **[CO_3_^2-^]** | 0.11 |
| **[DIC]** | 3.24 |
| **Ω_arag_** | 0.11 |

Table S5. Typical operating conditions for laser ablation ICP-MS analysis.

|  | **δ^11^B Isotope Analysis** | **Trace Element Analysis** |
| --- | --- | --- |
| **Instrument** |  |  |
| Mass Spectrometer | Thermo Scientific Neptune Plus multi-collector inductively coupled plasma mass spectrometer | Agilent 8900 Triple Quadrupole inductively coupled plasma mass spectrometer |
|  |  |  |
| Laser Ablation System | Elemental Scientific Lasers NWR193 excimer laser ablation system with a TwoVol2 ablation chamber | Elemental Scientific Lasers NWR193 excimer laser ablation system with a TwoVol2 ablation chamber |
|  |  |  |
| RF Power | 1400 W | 1550 W |
| Cones | Nickel skimmer (X) and jet sample | Standard nickel sample cone and XT skimmer |
|  |  |  |
| **Gas Flows** |  |  |
| Cooling Gas (argon) | 16 l min^-1^ | 13 l min^-1^ |
| Auxiliary Gas (argon) | 0.7 l min^-1^ | 0.56 l min^-1^ |
| Make-up gas (argon) | 1.0 l min^-1^ | 0.6 l min^-1^ |
| Ablation cell carrier gas (helium) | 0.7 l min^-1^ | 0.6 l min^-1^ |
| Additional Gas (nitrogen) | 0.005 l min^-1^ | 0.008 l min^-1^ |
|  |  |  |
|  |  |  |
| **Ablation Conditions** |  |  |
| Laser power density | ~6 J cm^-2^ | ~4.5 J cm^-2^ |
| Laser repetition rate | 20 Hz | 20 Hz |
| Laser beam size | 12 μm^-2^ to 60 μm^-2^ | 25 μm^-2^ |
| Laser tracking speed | 10 μm s^-1^ | 10 μm s^-1^ |
| Ablation mode | Line | Line |

Table S6. E/Ca analysis of in-house reference material PS69/318-1b by LA-ICP-MS

|  | **Li/Ca (μmol/mol)** | **B/Ca (μmol/mol)** | **Mg/Ca (mmol/mol)** | **Sr/Ca (mmol/mol)** | **Ba/Ca (μmol/mol)** | **U/Ca (nmol/mol)** |
| --- | --- | --- | --- | --- | --- | --- |
| **#1 ± 2SE** | 41.91 ± 1.46 | 225.71 ± 6.17 | 86.89 ± 0.79 | 3.27 ± 0.02 | 15.79 ± 0.39 | 17.87 ± 1.28 |
| **#2 ± 2SE** | 39.43 ± 1.25 | 221.85 ± 4.53 | 86.33 ± 1.16 | 3.15 ± 0.04 | 14.64 ± 0.43 | 16.58 ± 1.07 |
| **#3 ± 2SE** | 39.8 ± 1.36 | 221.29 ± 5.5 | 86.06 ± 1.07 | 3.15 ± 0.03 | 14.72 ± 0.18 | 16.95 ± 0.97 |
| **#4 ± 2SE** | 39.53 ± 1.49 | 223.42 ± 7.56 | 86.74 ± 0.86 | 3.16 ± 0.03 | 13.56 ± 1.04 | 17.96 ± 1.92 |
| **#5 ± 2SE** | 43.72 ± 1.9 | 226.27 ± 9.47 | 86.07 ± 0.96 | 3.15 ± 0.03 | 12.79 ± 0.67 | 18.87 ± 1.12 |
| **Mean ± 2SD** | 40.88 ± 3.77 | 223.71 ± 4.46 | 86.42 ± 0.76 | 3.18 ± 0.11 | 14.3 ± 2.31 | 17.65 ± 1.81 |

Table S7. Solution ICP-MS and MC-ICP-MS analysis of in-house reference material PS69/318-1b.

| **Ratio** | **Li/Ca (µmol/mol)** | **B/Ca (µmol/mol)** | | **Na/Ca (mmol/mol)** | | **Mg/Ca (mmol/mol)** |
| --- | --- | --- | --- | --- | --- | --- |
| **Mean ± 2SE** | 48.67 ± 10.79 | 233.82 ± 44.77 | | 26.23 ± 3.87 | | 78.97 ± 2.71 |
| **Ratio** | **Al/Ca (µmol/mol)** | **Mn/Ca (µmol/mol)** | | **Fe/Ca (µmol/mol)** | | **Sr/Ca (mmol/mol)** |
| **Mean ± 2SE** | 50.68 ± 2.92 | 1.43 ± 0.3 | | 19.9 ± 21.34 | | 2.9 ± 0.03 |
| **Ratio** | **Cd/Ca (nmol/mol)** | **Ba/Ca (µmol/mol)** | | **Nd/Ca (nmol/mol)** | | **U/Ca (nmol/mol)** |
| **Mean ± 2SE** | 192.87 ± 48.52 | 13.64 ± 3.41 | | 15.99 ± 7.69 | | 18.3 ± 1.67 |
| **Ratio** | **δ^11^B (‰)** |  |  | |  | |
| **Mean ± 2SE** | 15.25 ± 0.27 |  |  | |  | |
